# Design and application of a decatungstate-based ionic liquid photocatalyst for sustainable hydrogen atom transfer reactions†

**DOI:** 10.1039/d5gc02160j

**Published:** 2025-06-03

**Authors:** Miguel Claros, Julian Quévarec, Sara Fernández-García, Timothy Noël

**Affiliations:** a Flow Chemistry Group, Van't Hoff Institute for Molecular Sciences (HIMS), Universiteit van Amsterdam (UvA) 1098 XH Amsterdam The Netherlands t.noel@uva.nl; b Facultad de Química, Universidad de Murcia, Centro Multidisciplinar Pleiades-Vitalis Campus de Espinardo 30100 Murcia Spain

## Abstract

A recyclable decatungstate-based ionic liquid (DT-IL) was developed as a versatile photocatalyst for hydrogen atom transfer reactions. DT-IL exhibits broad solvent compatibility, high catalytic efficiency, and excellent recyclability. Its performance under batch and flow conditions, including in green and biphasic media, highlights its potential for sustainable photocatalysis.

## Abstract 2

Decatungstate-based ionic liquid photocatalyst (DT-IL) for sustainable hydrogen atom transfer reactions.

## Graphical abstract

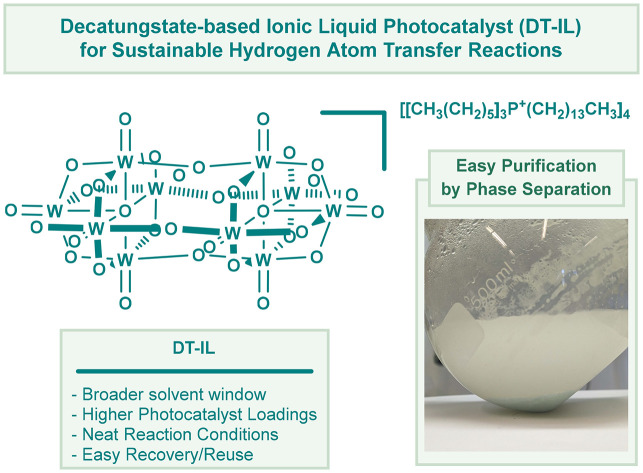

**Green foundation**

1. Catalysis: the decatungstate-based ionic liquid (DT-IL) functions as a recyclable photocatalyst, contributing to the sustainability of the transformation by reducing the need for fresh catalyst input in each cycle.

2. Waste prevention: the efficient recovery and reuse of DT-IL significantly minimize waste generation, offering a more sustainable alternative to conventional homogeneous photocatalysts that are typically discarded after use.

3. Safer solvents and reaction conditions: the ionic liquid nature of DT-IL broadens the range of compatible solvents and enables reactions at higher catalyst concentrations. This reduces the dependence on volatile or hazardous organic solvents, thereby enhancing both environmental safety and operational efficiency.

## Introduction

The promise of photocatalysis to enable more sustainable synthetic pathways through the use of light, rather than thermal energy, has led to a significant increase in research activity within the field of synthetic organic chemistry.^1^ To date, the majority of photocatalytic transformations have employed homogeneous photocatalysts, which, while effective, often suffer from limitations in terms of recyclability and separation. Heterogeneous photocatalysts have been developed as an alternative;^2^ however, their solid-state nature can result in complex light scattering and limited photon penetration into the reaction medium,^3^ thereby posing considerable challenges for the scale-up of such processes.^4^

While recycling of homogeneous photocatalysts is highly desirable, it is only feasible when the catalyst exhibits sufficient photostability. In many cases, the reactive radical species generated during photoredox processes can also interact with the photocatalyst itself, leading to its chemical modification and eventual degradation.^5^ Recently, we demonstrated that the hydrogen atom transfer (HAT) photocatalyst decatungstate anion ([W_10_O_32_]^4−^) exhibits exceptional robustness and can be efficiently recycled *via* nanofiltration without any observable decomposition.^6^ We attribute this stability to its inorganic molecular structure, composed exclusively of W–O bonds, which offers no obvious sites susceptible to radical attack.

While nanofiltration represents an attractive strategy for catalyst recovery in larger-scale applications,^7^ integrating this approach into small-scale, batch- or flow-type workflows remains challenging. In our pursuit of more sustainable and green photocatalytic alternatives, we questioned whether the common counterions of the decatungstate anion (*e.g.*, Na^+^ or tetrabutylammonium) could be exchanged to improve solvent compatibility. For instance, sodium decatungstate (NaDT) is typically used in highly polar solvents, whereas the tetrabutylammonium salt (TBADT) offers enhanced solubility in less polar media.^8^ However, its solubility remains largely restricted to solvents such as acetonitrile and acetone.^9^ Building on this foundation, we envisioned that pairing the decatungstate anion with bulkier organic cations—such as imidazolium or phosphonium—could offer enhanced tunability of key properties, including solubility, thermal stability, and catalytic performance,^10^ thus giving rise to ionic liquid derivatives of decatungstate (DT-ILs). These DT-ILs combine the robust photocatalytic activity of decatungstate with the inherent advantages of ionic liquids, such as thermal stability, low volatility, separability and high ionic conductivity, making them attractive candidates for a broad range of catalytic applications.^11^

To the best of our knowledge, DT-ILs were first reported by Kumar *et al*. in 2004, employing a 1-methyl-3-butylimidazolium cation in the high-temperature oxidation of alcohols to aldehydes or ketones.^12^ More recently, Floquet and co-workers described a phosphonium-based DT-IL that functioned as both solvent and catalyst in the oxidation of organic substrates.^13^ In their study, catalyst recyclability was achieved *via* size-exclusion chromatography; however, the reaction conditions also led to the irreversible formation of a catalytically inactive Lindqvist-type polyoxometalate, [W_6_O_19_]^2−^. Notably, both reports utilized DT-ILs in thermal oxidation reactions of alkanes and alcohols, whereas their potential as recyclable photocatalysts remains unexplored. In this work, we seek to address this gap by exploring the application of DT-ILs in photocatalytic processes, with a particular focus on hydrogen atom transfer (HAT) photocatalysis under continuous-flow conditions and their suitability for larger-scale implementation.

## Results

**Scheme 1:**
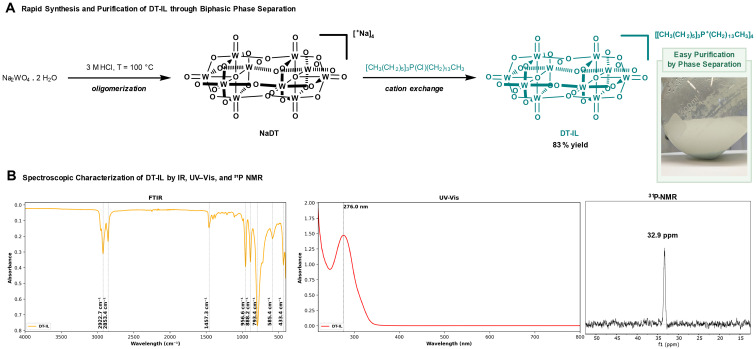
Synthesis and characterization of DT-IL.

We commenced our study by synthesizing the targeted decatungstate-based ionic liquid (DT-IL) (Scheme 1A). The procedure begins with the classical polymerization of sodium tungstate dihydrate under aqueous acidic conditions, initially forming sodium decatungstate (NaDT). This intermediate can be converted into the widely used tetrabutylammonium decatungstate (TBADT) *via* cation exchange with tetrabutylammonium bromide. At this stage, either NaDT or TBADT can be isolated through a labor-intensive sequence involving multiple washing, filtration, and drying steps. In contrast, ion exchange with trihexyltetradecylphosphonium chloride offers a significantly simpler and more efficient route. This method yields a dense, oily DT-IL phase that readily separates from the aqueous layer and can be dried under vacuum to give isolated yields of 83%, effectively eliminating many of the cumbersome steps associated with traditional Na^+^ or TBA^+^-based polyoxometalates. The entire process, including drying, can be completed within half a day—substantially shorter than the 3–5 days typically required to prepare TBADT in our laboratory.

The resulting DT-IL was characterized by infrared (IR) spectroscopy to confirm the integrity of the polyoxometalate structure (Scheme 1B, left). The band at 956 cm^−1^ corresponds to the apical W=O stretch, while those at 888, 793, and 586 cm^−1^ are assigned to W–O–W bridging vibrations. Additional bands in the regions 1377–1457 cm^−1^ and 2800–3100 cm^−1^ are attributed to aliphatic C–H vibrations of the organic cation. Furthermore, ^31^P-NMR spectroscopy provided insight into the phosphorus environment, enabling detection of the phosphonium cation and any residual phosphonium chloride from the synthesis (Scheme 1B, center). The characteristic phosphorus resonance also serves as a useful probe for monitoring the catalyst during reactions or extraction steps. The UV–Vis spectrum of DT-IL (Scheme 1B, right) closely resembles that of TBADT, exhibiting a broad absorption band in the near-UV region (250–350 nm) with a maximum at 280 nm, indicative of its suitability for photocatalytic applications.^14^

**Fig. 1:**
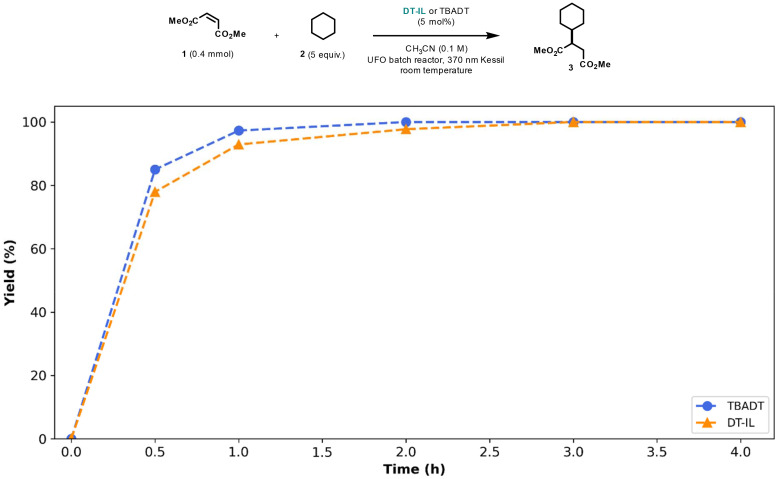
Kinetic profiles for the photocatalytic Giese-type coupling of cyclohexane and dimethyl maleate comparing TBADT and DT-IL as photocatalysts. Reaction conditions: TBADT or DT-IL (5 mol%), dimethyl maleate (0.4 mmol), cyclohexane (2.0 mmol), acetonitrile (0.1 M), UFO batch photochemical reactor,^16^ 370 nm Kessil LED (44 Watt).

The catalytic activity of DT-IL was evaluated using a Giese-type coupling reaction—a common benchmark in hydrogen atom transfer (HAT) catalysis involving the generation of a nucleophilic radical that subsequently adds to an electron-deficient olefin.^10,15^ In our initial trial, cyclohexane 2 was employed as the hydrogen donor and dimethyl maleate 1 as the radical acceptor (Fig. 1). Notably, a comparison between DT-IL and the conventional TBADT under identical conditions revealed comparable kinetic plots and full conversion after 1–2 hours of irradiation.

**Fig. 2:**
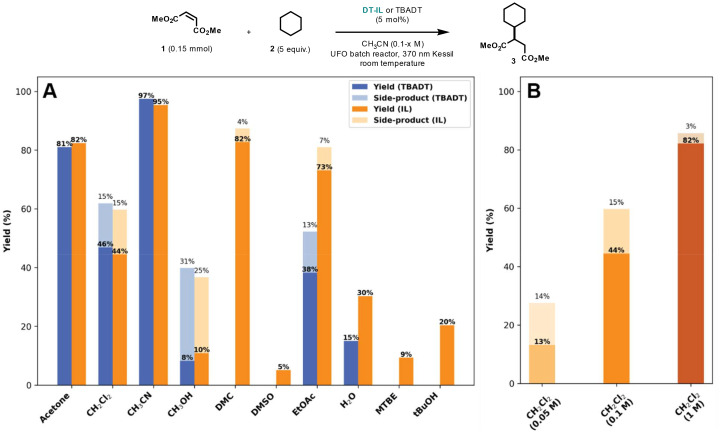
Yield comparison of the photocatalytic Giese-type coupling catalyzed by TBADT and DT-IL across different solvents (A), and at varying concentrations in dichloromethane (B). Side-products are derived from HAT activation of the solvent and its subsequent coupling with dimethyl maleate. Reaction conditions for solvent screening (A): TBADT or DT-IL (5 mol%), dimethyl maleate 1 (0.15 mmol), cyclohexane 2 (0.75 mmol), solvent (0.1 M), 2 h, UFO batch photochemical reactor, 370 nm Kessil LED (44 Watt). Reaction conditions for concentration screening (B): TBADT or DT-IL (5 mol%), dimethyl maleate 1 (0.15 mmol), cyclohexane 2 (2.0 mmol), dichloromethane (0.05–1 M), 2 h, UFO batch photochemical reactor, 370 nm Kessil LED (44 Watt).^16^

Building on the initial catalytic assessment, we next investigated the solubility profile of DT-IL to further optimize reaction conditions and enhance overall efficiency. In contrast to TBADT, which is predominantly soluble in acetonitrile or acetone, DT-IL exhibited moderate to good catalytic activity across a broader range of common organic solvents, including ethyl acetate (EtOAc), dimethyl carbonate (DMC), dichloromethane, and even water (Fig. 2A). However, diminished reactivity was observed in polar protic solvents such as methanol and ethanol, as well as in methyl *tert*-butyl ether (MTBE) and dimethyl sulfoxide (DMSO). Furthermore, DT-IL proved completely immiscible with nonpolar alkanes like pentane and heptane.

This broad solvent compatibility—particularly its excellent miscibility with polar aprotic solvents such as acetonitrile and acetone—facilitates practical deployment across diverse reaction setups. For example, concentrated stock solutions of DT-IL up to 0.12 M in acetonitrile could be readily prepared, representing catalyst loadings over 100-fold higher than those typically used for TBADT. In principle, even higher concentrations approaching the neat state are possible. Owing to its enhanced solubility, DT-IL allows higher catalyst loadings, which improves both reaction productivity and dosing accuracy, while also having a notable impact on selectivity. In TBADT-mediated Giese couplings, side products often arise from solvent activation pathways—particularly under dilute conditions where the substrate is present at low concentration. By enabling higher substrate and catalyst concentrations, DT-IL reduces the relative contribution of such undesired pathways, effectively suppressing side product formation and promoting selective C–C bond formation (Fig. 2B).

**Scheme 2:**
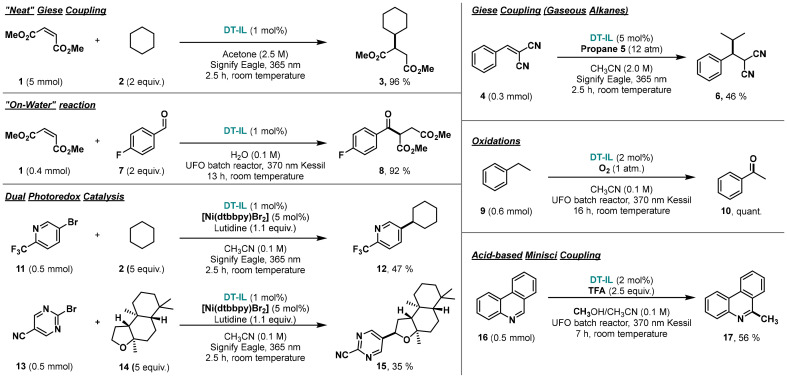
Catalytic versatility of DT-IL by applying it to a diverse set of hydrogen atom transfer (HAT)-type photocatalytic reactions. For experimental details, see ESI.†

These properties position DT-IL as a promising tool for automated, high-throughput reaction development, where the use of concentrated, stable stock solutions is critical.^17^ Additionally, DT-IL can function as both catalyst and solvent, enabling reactions to be conducted under near-neat conditions. To demonstrate this, we performed the model Giese coupling on a 5 mmol scale using only 2 equivalents of cyclohexane 2, which is significantly lower than the excess typically required for HAT processes. To facilitate mixing, 20% (v/v) acetone was added to reduce viscosity and improve homogeneity, as cyclohexane 2 and dimethyl maleate 1 are immiscible and DT-IL is a dense, viscous liquid. Under these conditions, the reaction proceeded efficiently, affording a 96% NMR yield for 3 (Scheme 2), consistent with results obtained under more dilute conditions.

Next, we aimed to explore the versatility of DT-IL by applying it to a diverse set of hydrogen atom transfer (HAT)-type photocatalytic reactions (Scheme 2). Due to its excellent solubility, DT-IL enabled the activation of more challenging gaseous alkanes.^18^ For example, by increasing the substrate concentration to 2 M, we successfully carried out the propanation of benzylidene malononitrile 4 within 2.5 hours using the Eagle photochemical flow reactor,^19^ achieving complete conversion (5, 56% isolated yield) with no detectable side products. This represents a significant advance, as the functionalization of gaseous alkanes is often limited by their low solubility and inherently lower reactivity compared to liquid substrates. Notably, conducting reactions at such high concentrations is uncommon in radical photochemistry, which is typically performed under dilute conditions to ensure photocatalyst solubility and optimal light penetration.

Although DT-IL is insoluble in water, it forms a stable emulsion after brief sonication at concentrations ranging from 1.6 to 3.2 mg mL^−1^ (0.00037–0.00075 M), which is within the effective range for decatungstate-mediated photocatalysis. To demonstrate its applicability in aqueous systems,^20^ we performed a direct “on water”^21^ hydroacylation of dimethyl maleate 1 using 4-fluorobenzaldehyde 7, which are two water-immiscible substrates at room temperature. The reaction proceeded smoothly, affording the acylated product 8 in 92% isolated yield.

DT-IL also performed well in photochemical oxidation reactions.^22^ The aerobic oxidation of ethylbenzene 9 to acetophenone 10 was completed in quantitative yield after 15 hours of irradiation in a UFO batch reactor.^16^

To further highlight the versatility of the system, we evaluated DT-IL in a dual catalysis setup combining HAT activation with nickel-catalyzed cross-coupling. In this dual photocatalytic transformation, the alkylation of 5-bromo-2-(trifluoromethyl)pyridine 11 with cyclohexane 2 proceeded with 47% yield after only 15 minutes of residence time in an Eagle flow reactor.^23^ Similarly, ambroxide 14 was successfully arylated, affording the cross-coupled product 15 in 35% isolated yield. Notably, the ionic nature of DT-IL facilitated simple product purification. The arylated product was readily extracted using pentane, while nickel residues and other impurities remained in the DT-IL/acetonitrile phase. The catalyst was subsequently recovered by dissolving the crude reaction mixture in ethyl acetate and washing with aqueous HCl to remove metal residues. After solvent removal, DT-IL was fully recovered and reused without loss of performance.

The solubility and stability of DT-IL across a variety of solvents provided a robust platform for exploring diverse photocatalytic transformations, enabling efficient reaction conditions and broad substrate compatibility. For example, a green solvent mixture of acetonitrile and methanol proved effective for a Minisci-type alkylation of heteroarenes (16), with methanol serving as both co-solvent and alkyl source (Scheme 2).^24^

Beyond its remarkable catalytic versatility, the most significant advantage of the decatungstate-based ionic liquid (DT-IL) lies in its exceptional recyclability, making it particularly attractive for sustainable photocatalysis. Following each photocatalytic reaction, DT-IL was readily recovered through simple solvent extraction and reused in subsequent reactions without notable loss of activity. It consistently retained high catalytic efficiency over multiple cycles, showing minimal degradation and maintaining performance even after several rounds of reuse.

This combination of stability and ease of recovery positions DT-IL as a promising candidate for large-scale applications, where both economic and environmental factors are critical. To this end, several recycling protocols were employed over the course of this study.^25^ Initially, a straightforward pentane extraction from the acetonitrile-based reaction medium proved effective for isolating products from non-polar substrates. However, this approach was less efficient with more polar substrates, necessitating alternative strategies (see ESI†).

**Scheme 3:**
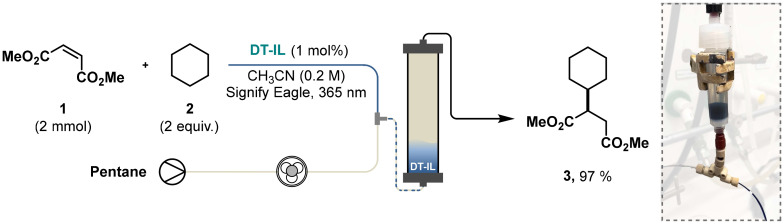
Continuous extraction of DT-IL with pentane enables effective recovery of the photocatalyst and product 3 purification.

A particularly effective method involved a continuous extraction setup (Scheme 3). In this protocol, the crude reaction mixture exiting the reactor was passed through an empty polypropylene chromatography cartridge, while pentane was continuously introduced from bottom to top. This process enabled selective extraction of the coupling product 3 (97% isolated yield), leaving behind the DT-IL as a dense, oily residue. Extraction progress could be monitored by sampling the pentane stream for GC-MS analysis, or, in the case of UV-active products, using an inline UV–Vis detector to track the product's elution. Using this method, we demonstrated that the catalyst could be recycled over at least five consecutive cycles, consistently delivering the coupling product 3 in excellent yield.

## Conclusions

In this study, we have introduced a recyclable decatungstate-based ionic liquid (DT-IL) that addresses key limitations of traditional homogeneous photocatalysts in hydrogen atom transfer (HAT) chemistry. By combining the catalytic robustness of decatungstate with the tunability of ionic liquids, DT-IL exhibits excellent solubility, thermal stability, and compatibility with a broad solvent range. This unique solubility profile enables highly concentrated reaction setups, improving both reaction efficiency and selectivity. DT-IL proved effective in a wide array of transformations, including Giese-type couplings, Minisci reactions, aerobic oxidations, and dual photocatalytic cross-couplings. Importantly, its ionic nature allows for straightforward separation from reaction mixtures and efficient recovery *via* practical batch or continuous extraction protocols. The catalyst retained high activity over multiple cycles, demonstrating minimal degradation and operational flexibility in both batch and flow systems. These features collectively establish DT-IL as a promising tool for sustainable photocatalysis, with strong potential for adoption in high-throughput, scalable chemical manufacturing.

## Author contributions

The roles as defined by CRediT for this work are as follows: conceptualization (M. C., T. N.), data curation (M. C., J. Q., S. F. G.), funding acquisition (T. N.), investigation and methodology (M. C., J. Q., S. F. G.), project administration (M. C., T. N.), supervision (M. C., T. N.), writing – original draft writing (M. C., J. Q.) – review & editing (T. N. with input from all authors).

## Conflicts of interest

The authors declare no conflict of interest.

## Supplementary Material

GC-027-D5GC02160J-s001

## Data Availability

Experimental procedures, spectroscopic characterization and analytic methods are available in the ESI.†

## References

[cit1] Noël T., Zysman-Colman E. (2021). Chem. Catal..

[cit2] Gisbertz S., Pieber B. (2020). ChemPhotoChem.

[cit3] Zondag S. D. A., Schuurmans J. H. A., Chaudhuri A., Visser R. P. L., Soares C., Padoin N., Kuijpers K. P. L., Dorbec M., van der Schaaf J., Noël T. (2024). Nat. Chem. Eng..

[cit4] Zondag S. D. A., Mazzarella D., Noël T. (2023). Annu. Rev. Chem. Biomol. Eng..

[cit5] Devery III J. J., Douglas J. J., Nguyen J. D., Cole K. P., Flowers II R. A., Stephenson C. R. J. (2015). Chem. Sci..

[cit6] Wen Z., Pintossi D., Nuño M., Noël T. (2022). Nat. Commun..

[cit7] Dijkstra H. P., van Klink G. P. M., van Koten G. (2002). Acc. Chem. Res..

[cit8] Capaldo L., Ravelli D., Fagnoni M. (2022). Chem. Rev..

[cit9] Singh P. P., Sinha S., Gahtori P., Tivari S., Srivastava V. (2024). Org. Biomol. Chem..

[cit10] Martinetto Y., Pégot B., Roch-Marchal C., Haouas M., Cottyn-Boitte B., Camerel F., Jeftic J., Morineau D., Magnier E., Floquet S. (2021). New J. Chem..

[cit11] Dupont J., Leal B. C., Lozano P., Monteiro A. L., Migowski P., Scholten J. D. (2024). Chem. Rev..

[cit12] Chhikara B. S., Tehlan S., Kumar A. (2005). Synlett.

[cit13] Martinetto Y., Basset S., Pégot B., Roch-Marchal C., Camerel F., Jeftic J., Cottyn-Boitte B., Magnier E., Floquet S. (2021). Molecules.

[cit14] Hong B., Indurmuddam R. R. (2024). Org. Biomol. Chem..

[cit15] Ravelli D., Fagnoni M., Fukuyama T., Nishikawa T., Ryu I. (2018). ACS Catal..

[cit16] Masson T. M., Zondag S. D. A., Schuurmans J. H. A., Noël T. (2024). React. Chem. Eng..

[cit17] Slattery A., Wen Z., Tenblad P., Sanjosé-Orduna J., Pintossi D., den Hartog T., Noël T. (2024). Science.

[cit18] Laudadio G., Deng Y., van der Wal K., Ravelli D., Nuño M., Fagnoni M., Guthrie D., Sun Y., Noël T. (2020). Science.

[cit19] Wan T., Wen Z., Laudadio G., Capaldo L., Lammers R., Rincón J. A., García-Losada P., Mateos C., Frederick M. O., Broersma R., Noël T. (2022). ACS Cent. Sci..

[cit20] Kitanosono T., Masuda K., Xu P., Kobayashi S. (2018). Chem. Rev..

[cit21] Chatgilialoglu C., Barata-Vallejo S., Gimisis T. (2024). Molecules.

[cit22] Laudadio G., Govaerts S., Wang Y., Ravelli D., Koolman H. F., Fagnoni M., Djuric S. W., Noël T. (2018). Angew. Chem., Int. Ed..

[cit23] Mazzarella D., Pulcinella A., Bovy L., Broersma R., Noël T. (2021). Angew. Chem., Int. Ed..

[cit24] McCallum T., Pitre S. P., Morin M., Scaiano J. C., Barriault L. (2017). Chem. Sci..

[cit25] Vural Gürsel I., Noël T., Wang Q., Hessel V. (2015). Green Chem..

