# Supplementary material for: Design and application of a decatungstate-based ionic liquid photocatalyst for sustainable hydrogen atom transfer reactions
Source: Green Chem. 2025 Jun 3;27(25):7660–6. doi: 10.1039/d5gc02160j (PMC12143237; doi:10.1039/d5gc02160j)
Supplement: GC-027-D5GC02160J-s001 [file GC-027-D5GC02160J-s001.pdf]

Supplementary Information (SI) for Green Chemistry.
This journal is © The Royal Society of Chemistry 2025

Design and Application of a Decatungstate-based Ionic Liquid Photocatalyst for Sustainable Hydrogen Atom Transfer Reactions

Supplementary Information

Miguel Claros,[a†] Julian Quévarec,[a †] Sara Fernández-García,[b] Timothy Noël,[a*]

[1] Flow Chemistry Group, Van’t Hoff Institute for Molecular Sciences (HIMS), Universiteit van Amsterdam (UvA), 1098 XH Amsterdam, The Netherlands.

[2] Facultad de Química, Universidad de Murcia, Centro Multidisciplinar Pleiades-Vitalis, Campus de Espinardo, 30100 Murcia, Spain.

[†] These authors contributed equally.

* Corresponding Author: t.noel@uva.nl (Timothy Noël)

Contents

1. General Information 3
2. Reactor Design 4
   2.1 Flow Equipment 5
   2.2 Signify Eagle Reactor 5
   2.3 Photochemical Set-Up for Gas-Liquid reactions. 6
   2.4 DT-IL Recovery Set-up 6
3. DT-IL Catalyst Preparation 6
4. Photocatalytic Reactivities 8
5. Continuous Extraction Procedure 14
6. Other Recycling Attempts 16
7. Kinetic Experiments 18
8. Solvent Screening 19
9. E Factor Calculations 21
10. References 24
11. Spectra 24

1. General Information

Reagents and consumables. All reagents and solvents were bought from Sigma Aldrich, TCI, Fluorochem, VWR International and Biosolv and used as received. Disposable syringes were purchased from Laboratory Glass Specialist. Product isolation was performed manually, using silica gel (60, F254, Merck™), or automatically, by a Biotage Isolera One Flash automated chromatography system. TLC analysis was performed using Silica on aluminum foils TLC plates (F254, Supelco Sigma-Aldrich™) with visualization under ultraviolet light (254 nm and 365 nm) or TLC staining (KMnO4 or vanillin).

NMR spectroscopy. ^{1}H (400 MHz or 300 MHz), ^{13}C (101 MHz or 75 MHz) and ^{19}F (376 MHz or 282 MHz) spectra were recorded unless stated otherwise on ambient temperature using a Bruker AV400 or a Bruker AV300. ^{1}H-NMR spectra are reported in parts per million (ppm) downfield relative to $CDCl_3$ (7.26 ppm), ^{13}C-NMR spectra are reported in ppm relative to $CDCl_3$ (77.2 ppm), ^{19}F-NMR spectra are reported in ppm relative to PhF (-113.15 ppm) and ^{31}P-NMR spectra were measured without ^{31}P internal standard. The multiplicities of signals are designated by the following abbreviations: s (singlet), d (doublet), t (triplet), q (quartet), m (multiplet), dd (doublet of doublets), dt (doublet of triplets), td (triplet of doublets), tt (triplets of triplets), ddd (doublet of doublet of doublets), qd (quartet of doublet). Coupling constants (J) are reported in hertz (Hz). NMR data was processed using the MestReNova 14 software package. Known products were characterized by comparing ^{1}H NMR and ^{13}C NMR spectra with those available in the literature.

GC-MS Spectroscopy. GC-MS analysis was performed on an Agilent 8890 gas chromatograph coupled with an Agilent 5977C mass detector.

UV-Vis spectroscopy. UV-Vis spectra were recorded with a single-beam Duetta ExSpec equipped with a Xe arc lamp (250-1000 nm) and a CCD camera as a detector. Measurements were performed in a quartz cuvette (optical path: 1 cm). For quantum yield measurements, a double beam spectrophotometer Shimadzu UV2700 equipped with a deuterium lamp (190-350 nm), a halogen lamp (330-900 nm) and a photomultiplier (Hamamatsu R928).

IR spectroscopy. IR spectra were recorded on a Bruker Alpha FTIR apparatus equipped with a high-resolution DTGS detector and an air-cooled 12V, 20W IR source, operating in the 400–4000 cm^{-1} range with a spectral resolution of 2 cm^{-1}. Measurements were performed in reflection mode using a gold reference cap, with a sampling spot diameter of 5 mm.

2. Reactor Design

Batch photoreactions were performed in glass tubes (7.5 mL, 13 × 100 mm, Pyrex, Corning) using the 3d printed "UFO" photoreactor described by Masson et al.,[1] and a Gen2 370nm Kessil lamp was used as the light source.

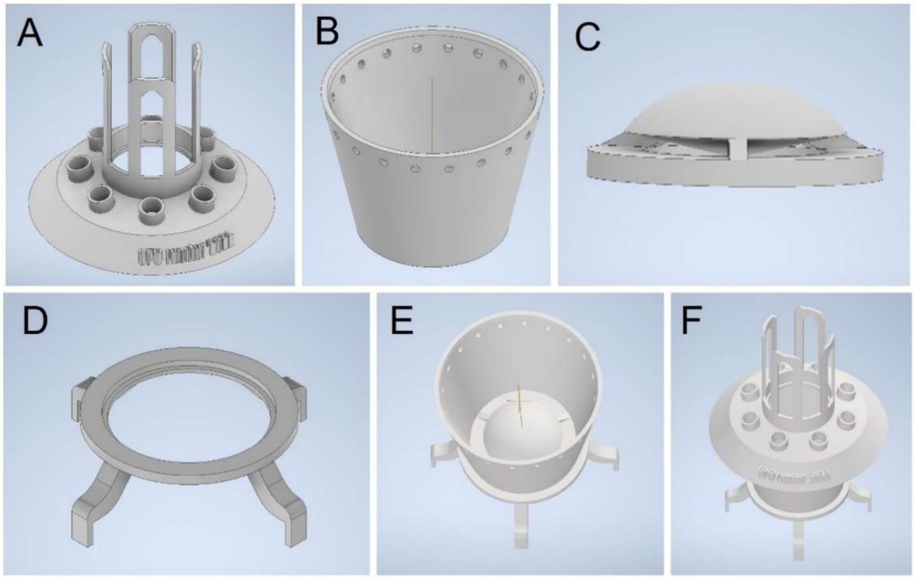

Figure S1. *Overview of the 3D-printed reactor: A) lid designed to host up to 8 reactions vials and hold the Kessil lamp in the center; B) body of the reactor; C) light reflector: it is coated with reflective tape; D) adapter for stirring plate; E) inside of the reactor; G) overall reactor.*

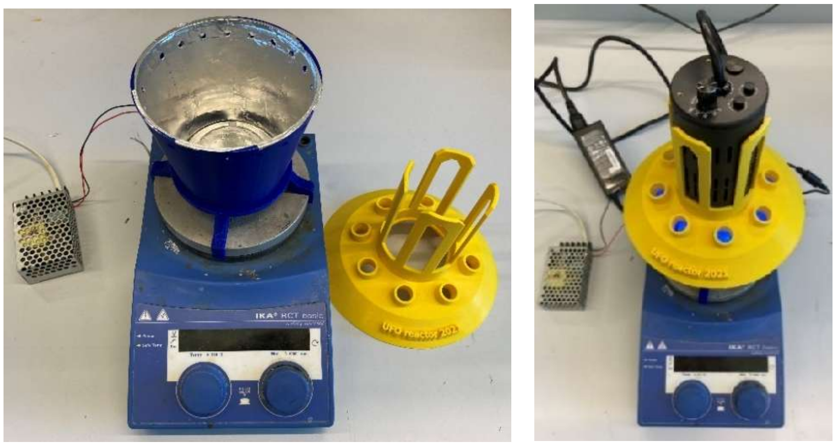

Figure S2*. Pictures of the assembled reactor equipped with a Kessil lamp (365 nm).*

Flow photoreactions were performed in the Signify Eagle reactor designed by Signify in collaboration with our team using a Shimadzu LC-40D HPLC pump.[2] The flow recycling procedure was performed in Screening Devices with empty chromatography cartridges. Flow connections and tubing were purchased from Screening Devices.

2.1 Flow Equipment

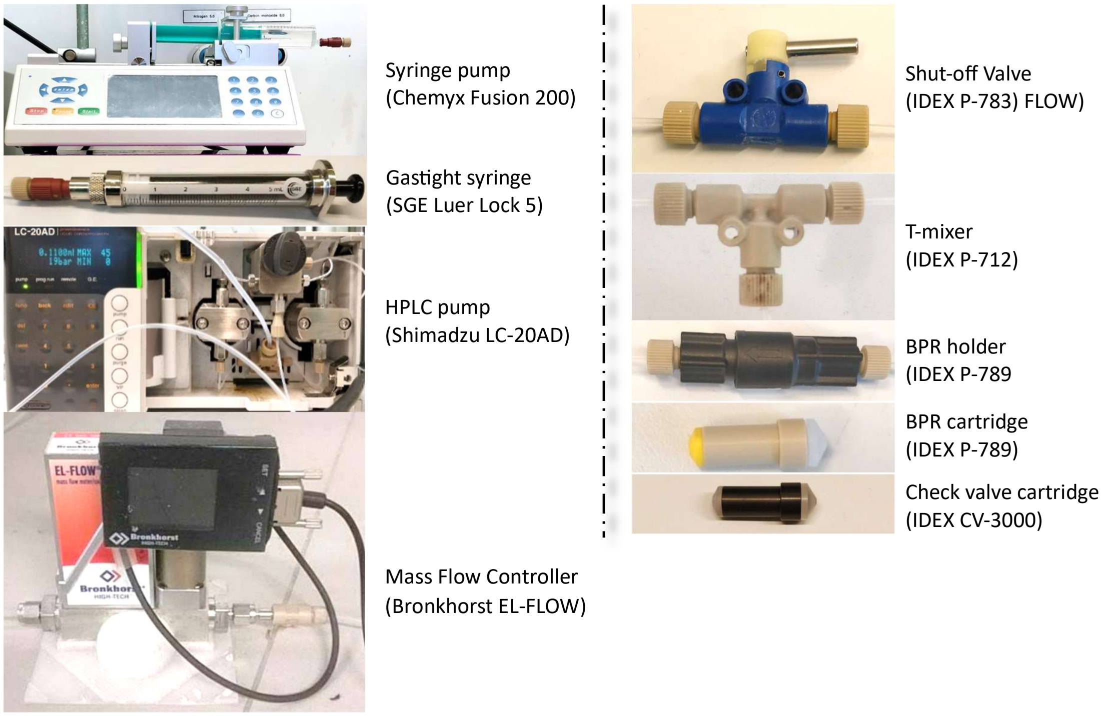

Figure S3. *Details of flow equipment used for the photocatalytic reactions*.

2.2 Signify Eagle Reactor

A Signify Eagle photochemical reactor was used, consisting of a base assembly with six 365 nm UV-A chip-on-board light modules. Each of these light source modules contain a fan and a heat sink to efficiently dissipate heat generated through the high power LEDs. Also, the head cap assembly contains blowers to cool the interior of the reactor system, to reduce undesired thermal side-reactions. The LED modules and chamber cooling blowers are connected to a driver box, allowing to set the current of each of the LED modules individually, as well as the rotation speed of the cooling blowers. The six LED modules (365 nm, max. 144 W combined optical output power) are positioned in a hexagonal form around an aluminium cylinder support (80 mm height, 75 mm diameter), which has the reactor coil wrapped around (FEP capillary tubing: 0.8 mm ID, 4 mL volume).

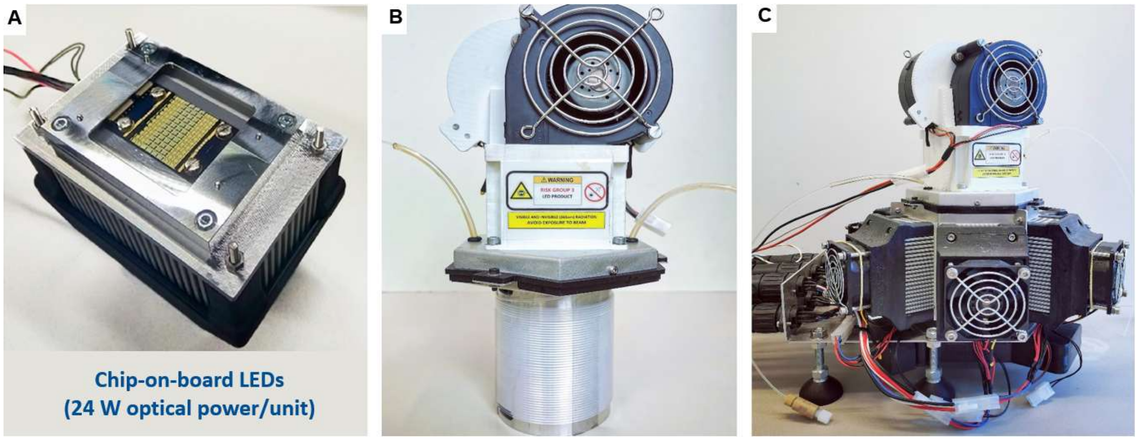

Figure S4. *Signify Eagle Reactor with six (A) chip-on-board LED modules, (B) head assembly with reactor coil, and (C) complete assembly with fans, heat sinks and LED modules.*

2.3 Photochemical Set-Up for Gas-Liquid reactions.

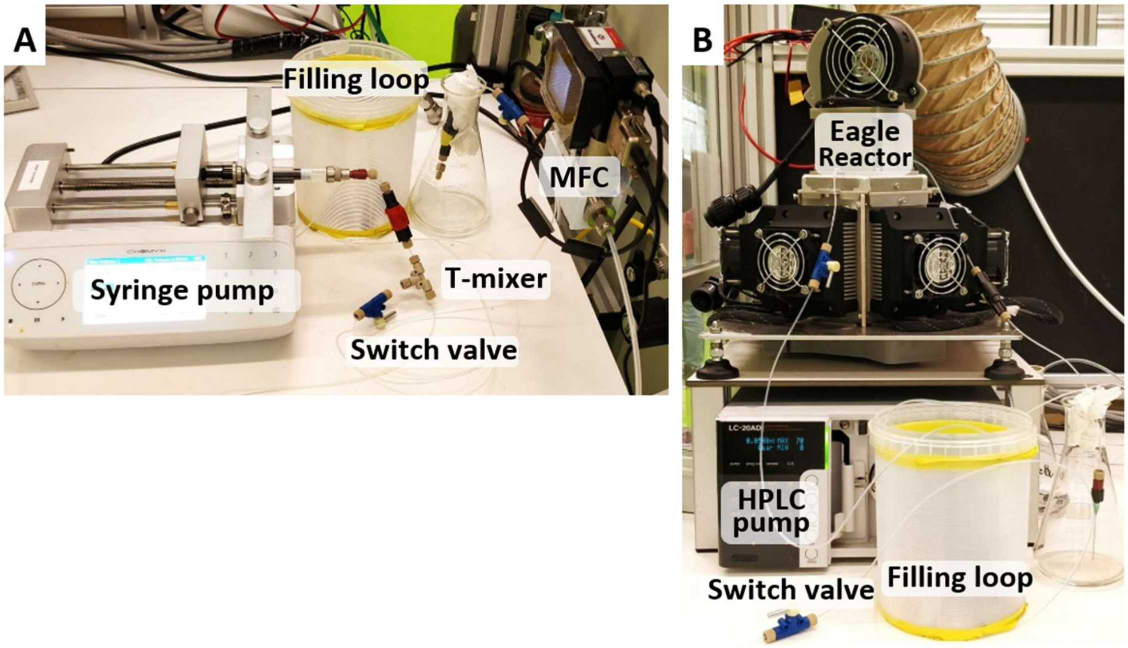

Figure S5. *Overview of Setup for (A) loop filling of gas- and liquid, (B) photochemical reaction with Signify Eagle Reactor.*

2.4 DT-IL Recovery Set-up

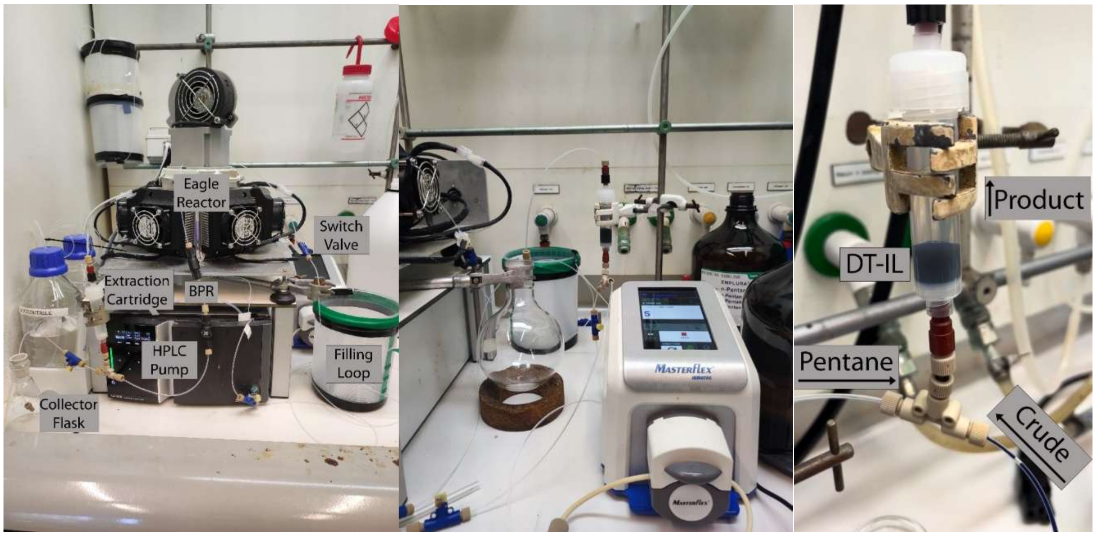

Figure S6. *Experimental Set-up for the inflow extraction procedure.*

3. DT-IL Catalyst Preparation

In a 250mL Erlenmeyer flask, sodium tungstate dihydrate (16 g, 50 mmol) was dissolved in boiling deionized water (100 mL). Boiling hydrochloric acid (33.5 mL, 3 M, 100 mmol, 2 equiv.) was added in

one portion to the tungstate solution with vigorous stirring. After 2 minutes of strong boiling, a solution of P[6,6,6,14]Cl (7.55 g, 15 mmol, 0.3 equiv.) in 10 mL ethanol was added, heating was stopped, and the mixture was allowed to cool down to room temperature. The POM-IL forms a dense phase at the bottom of the flask, the aqueous phase was removed, and the IL phase was washed three times with boiling deionized water, evaporated under vacuum in a rotary evaporator, and dried under vacuum to afford a dense blue-green oil. Yield: 13.4 g (3.12 mmol, 62 % yield based on the tungstate, 83 % yield based on the limiting reagent).

^{31}P-NMR (121 MHz, $CDCl_3$) δ = 32.9 (m) ppm.

IR (cm-1) 2953, 2923, 2853, 1457, 1408, 1377, 1111, 956, 889, 793, 714, 585, 433, 402.

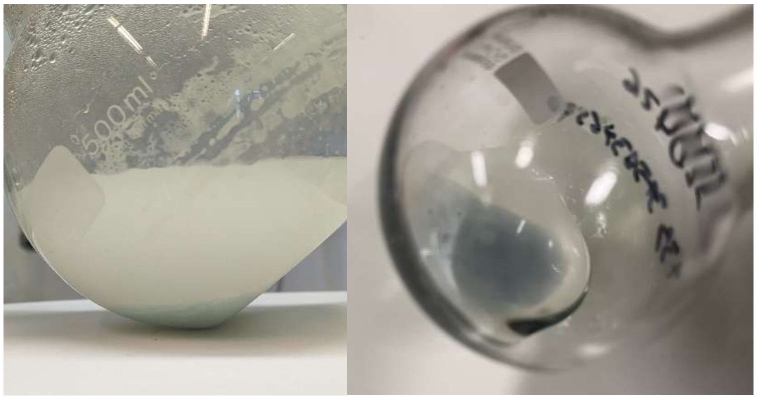

Figure S7. *DT-IL oiling out during the preparation (left) and after being dried under high vacuum (right).*

UV-Vis spectra

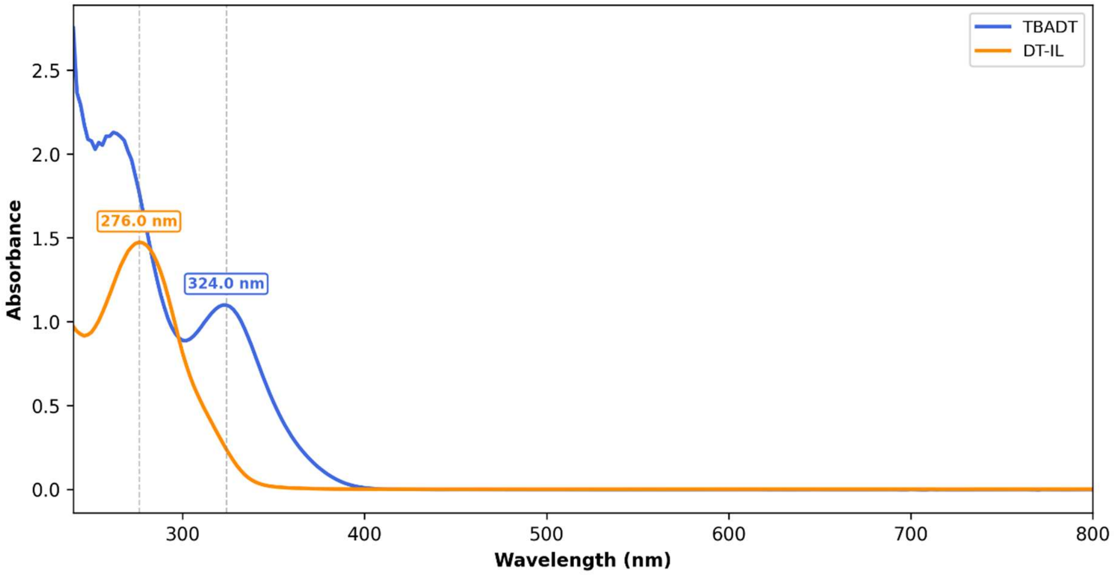

Figure S8. *UV-Vis spectra of TBADT (Blue) and DT-IL (orange) at 0.1 mM in acetonitrile.*

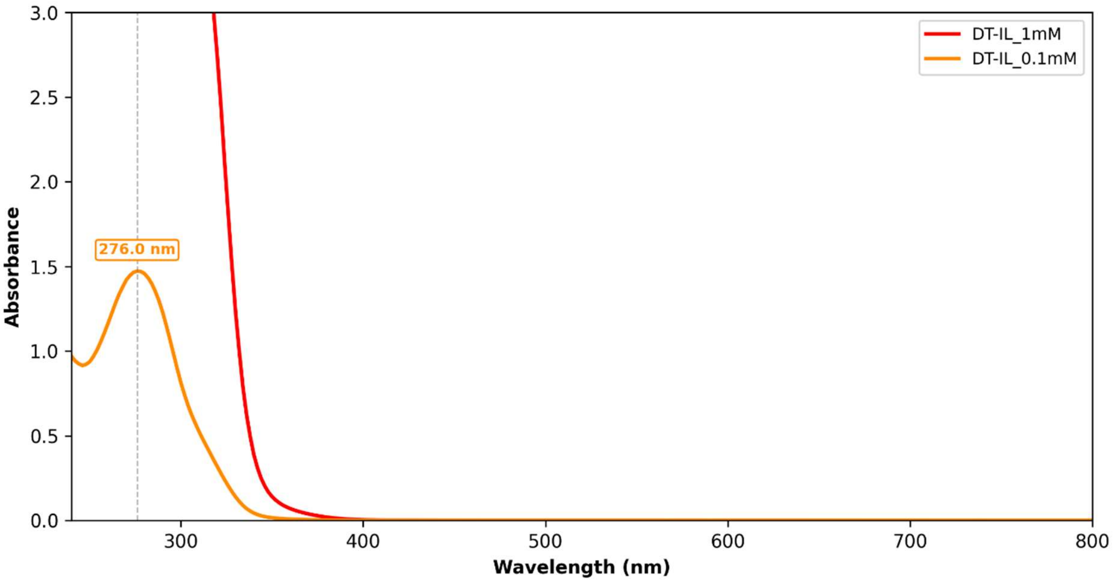

Figure S9. *UV-Vis spectra of DT-IL 0.1 mM (orange) and DT-IL 1 mM (red) in acetonitrile.*

A comparison of the UV-Vis spectra of TBADT and DT-IL reveals a blue shift in the absorbance maximum for DT-IL. While the absorbance around 365 nm is negligible at a concentration of 0.1 mM, at the concentration used for photocatalytic experiments (1 mM), the ionic liquid exhibits noticeable absorbance in this region.

4. Photocatalytic Reactivities

Giese Coupling.

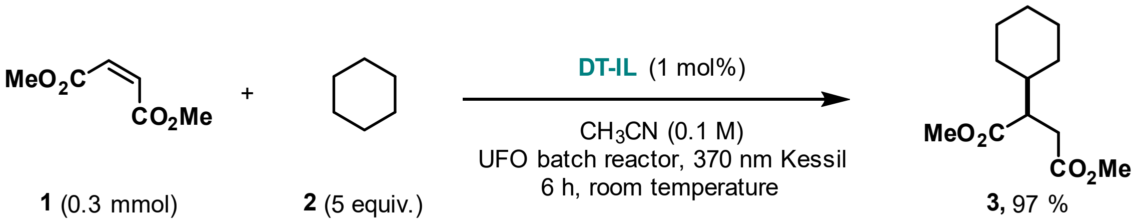

Acetonitrile (3 mL) was added to P[6,6,6,14]DT (12.9 mg, 0.003 mmol, 1 mol%) in a 7.5 mL glass vial, and the mixture was sonicated until complete dissolution of the catalyst (1 min). Dimethyl maleate (**1**, 37 mL, 0.3 mmol, 1 equiv.) and cyclohexane (**2**, 160 mL, 1.5mmol, 5equiv.) were added to the vial along with a stir bar. The mixture was shaken and introduced in the “UFO” reactor. After six hours, biphenyl (1 equiv.) was added as an internal standard for quantitative GC analysis, or the mixture was filtered through a silica-filled Pasteur pipette and evaporated to afford the crude product. The product was isolated by column chromatography on silica gel using 9/1 Pentane/EtOAc as the eluent.

^{1}H-NMR (300 MHz, $CDCl_3$): δ = 3.69 (s, 3H, CH_3), 3.66 (s, 3H, CH_3), 2.72 (m, 2H, CH_2), 2.45 (dt, J = 8.9Hz and 13.4Hz, 1H, CH), 1.70-1.79 (m, 2H, CH_2), 1.53-1.69 (m, 4H, 2x CH_2), 1.14-1.31 (m, 2H, CH_2), 0.96-1.14 (m, 3H, 1 CH + CH_2) ppm.

The NMR is in accordance with the literature.[3]

Giese Coupling under "neat" conditions.

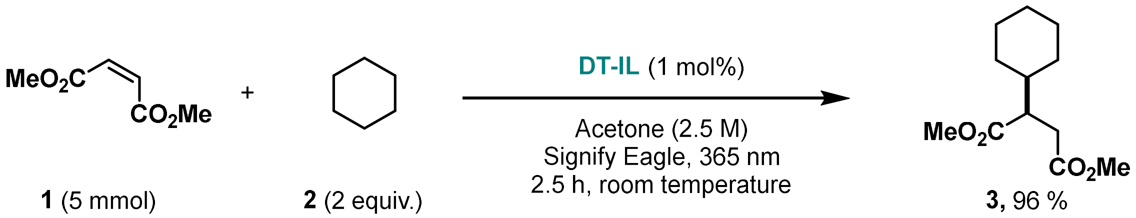

1 (5 mmol) **2** (2 equiv.) **3,** 96 %

Dimethyl maleate (**1**,627 µL, 5 mmol, 1 equiv.) was added to P[6,6,6,14]DT (214 mg, 0.05 mmol, 1 mol%) in a 7.5 mL glass vial and the mixture was sonicated until homogenous (~2 min). Cyclohexane (**2**, 1.09 mL, 10 mmol, 2 eq) was added to the vial, forming two phases. Acetone (300 µL) was added, and the mixture was shaken until homogeneous. The reaction mixture was injected in a pre-loop* using a 3 mL syringe and was pumped inside an eagle reactor set to 144 W using an HPLC pump at a flow rate of 0.02 mL/min in a 3 mL reactor to get a residence time of 2.5 h. The conversion of the reaction was determined by ^{1}H-NMR obtaining 96 % of yield for the desired product. Control experiments were performed to confirm that the DT-IL catalyst is required for the reaction to occur. In the absence of the catalyst, no product formation was observed, and the starting material was fully recovered, ruling out any activation by acetone.

*A pre loop/ HPLC pump system was used here to limit the loss of reaction mixture compared to using a syringe/syringe pump system, as the latter would require changing the initial syringe to a solvent syringe when the reaction mixture has been injected to finish flowing the mixture through the reactor coil.

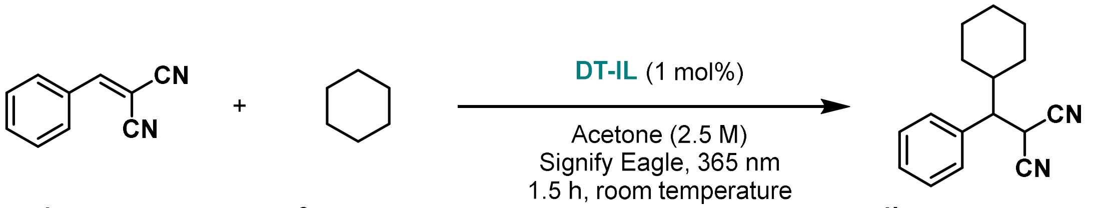

4 (5 mmol) **2** (2 equiv.) **4b,** 73 % NMR-Yield

Benzylidene malonitrile (**4**, 771 mg, 5 mmol, 1 equiv.) was added to P[6,6,6,14]DT (214 mg, 0.05 mmol, 1 mol%) in a 7.5 mL glass vial and the mixture was sonicated until homogenous (~2 min). Cyclohexane (**2**, 1.09 mL, 10 mmol, 2 eq) was added to the vial, forming two phases. Acetone (400 µL) was added, and the mixture was shaken until homogeneous. The reaction mixture was injected in a pre-loop*

using a 3 mL syringe and was pumped inside an eagle reactor set to 144 W using an HPLC pump at a flow rate of 0.03 mL/min in a 3 mL reactor to get a residence time of 1.5 h. The conversion of the reaction was determined by ^{1}H-NMR obtaining 74 % of yield for the desired product.

Giese Coupling with light alkanes.

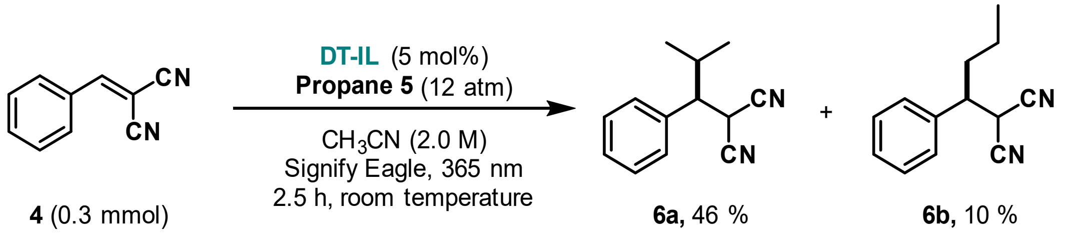

A nitrogen-purged, screw-capped vial, fitted with a rubber septum was charged with 2-benzylidenemalononitrile (**4**, 2.0 mmol, 308 mg) and DT-IL (5 mol%, 0.1 mmol, 429 mg) and dry CH_3CN (1.1 mL). The mixture was sonicated until homogeneity and charged in a 3 mL gastight syringe, positioned in a syringe pump and combined with a stream of propane gas (**5**, 10 mmol, 5 equiv.) through a T-mixer into a filling loop, with a liquid flow rate of 0.02 mL·min-1 and a propane gas flow rate of 4.69 mL·min-1. A BPR of 2.8 bar was used during the loop filling. Next, the filling loop was connected to the reactor, the system was pressurized to 12 bar using an HPLC pump and the reaction mixture was pumped over the Signify Eagle reactor (365 nm, 144 W output power, FEP capillary: 0.5 mm ID, 2.7 mL) at a flow rate of 0.02 mL·min-1, resulting in a residence time of 2.5 h. The obtained reaction mixture was collected into cartridge and extracted inline by pumping 500 mL of pentane by a peristaltic pump (see figure S6 for the set-up). The product was obtained as a pure mixture of the branched (**6a**) and linear (**6b**) products (83:17).

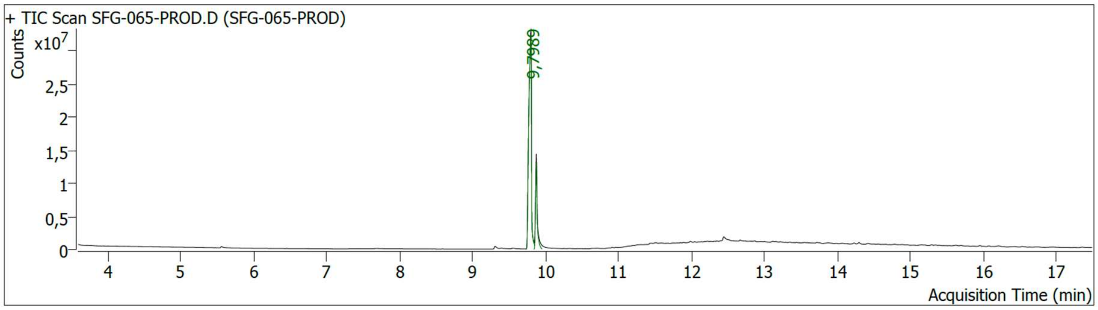

Figure S10. *Chromatogram of the solution after extraction with pentane.*

^{1}H-NMR (300 MHz, $CDCl_3$): δ = 7.47 – 7.39 (m, 3H), 7.34 (dd, *J* = 7.5, 2.1 Hz, 2H), 4.18 (d, *J* = 5.6 Hz, 1H), 2.87 (dd, *J* = 9.7, 5.6 Hz, 1H), 1.17 (d, *J* = 6.6 Hz, 3H), 0.86 (d, *J* = 6.6 Hz, 3H) ppm.

The NMR is in accordance with the literature.[4]

"On-Water" Reaction.

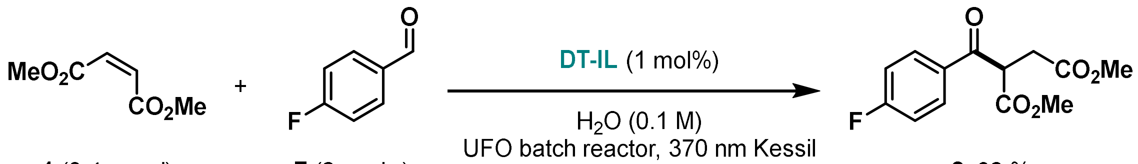

Dimethyl maleate (**1**, 50 µL, 0.4 mmol, 1equiv.) and 4-fluorobenzaldehyde (**7**, 86 µL, 0.8 mmol, 2 equiv.) were added to P[6,6,6,14]DT (13.6 mg, 0.003 mmol, 0.7 mol%) in a 20 mL scintillation vial. The mixture was sonicated and shaken until homogenous (~2 min). Water (4 mL, 0.1 M) was added to the stock solution, and the mixture was shaken. The solution (4 mL) described above was introduced in a 7.5mL glass vial with a stirring bar, and the vial was introduced in the "UFO" reactor for 13 h. The reaction mixture was extracted four times with dichloromethane. The organic layers were combined and dried over anhydrous sodium sulfate. The crude product was purified by column chromatography (10% EtOAc in pentane). Yield: 94mg (0.35mmol, 92%) of a clear-yellow oil.

^{1}H NMR (300 MHz, $CDCl_3$): δ = 8.08 (m, 2H, 2x $CH_{aromatic}$), 7.17 (m, 2H, 2x $CH_{aromatic}$), 4.84 (dd J = 6.5Hz and 8.0Hz, 1H, CH), 3.69 (s, 3H, CH_3), 3.68 (s, 3H, CH_3), 3.08 (m, 2H, CH_2) ppm.
^{19}F NMR (282 MHz, $CDCl_3$): δ = -103.92 (m) ppm.
The NMR is in accordance with the literature.[5]

Determination of the catalyst concentration in water emulsion:

P[6,6,6,14]DT (8.6 mg, 0.002 mmol) was introduced in a 5 mL glass vial, deionized water (1 mL) was added, and the mixture was sonicated and shaken until the formation of an emulsion. The mixture was allowed to rest for 10 min, and 500 µL of the emulsion was transferred to another vial. The aliquot was evaporated under a strong vacuum until a constant mass was measured. The amount of catalyst collected was between 0.8 mg and 1.6mg, corresponding to a concentration ranging between 1.6 mg/mL and 3.2 mg/mL (from 0,00037 M to 0,00075 M).

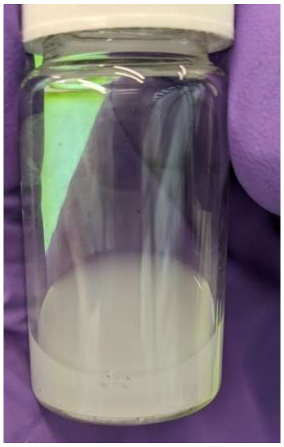

Figure S11. *Stable emulsion of the DT-IL on water.*

Oxidation of Ethyl benzene

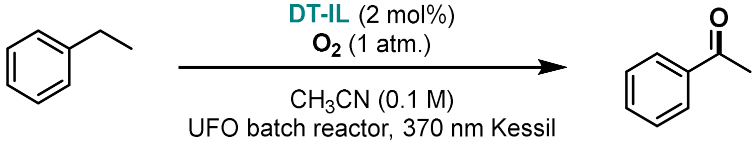

9 (0.6 mmol) **10**, quant.

Acetonitrile (1.4 mL) was added to DT-IL (17 mg, 0.004 mmol, 2 mol%) in a 7.5 mL glass vial (UFO vial) containing a stir bar. The vial was sonicated to ensure complete dissolution. Ethylbenzene (**9**, 24.5 µL, 0.2 mmol, 1 equiv.) was added followed by 1 M HCl (0.6 mL), the mixture was shaken before bubbling O_2 through it with a balloon for 1 min. The vial was then placed in a UFO reactor and irradiated at 365 nm for 15 h. The reactor mixture was diluted with water and extracted with EtOAc. The organic extracts were combined and dried over magnesium sulphate, and evaporated under reduced pressure.

^{1}H NMR (300 MHz, $CDCl_3$): 7.95 (d, J = 7.0 Hz, 2H), 7.60 – 7.51 (m, 1H), 7.44 (td, J = 7.0, 6.2, 1.3 Hz, 2H), 2.58 (d, J = 0.8 Hz, 3H)ppm.

The NMR is in accordance with the literature.[6]

Dual Photoredox Catalysis

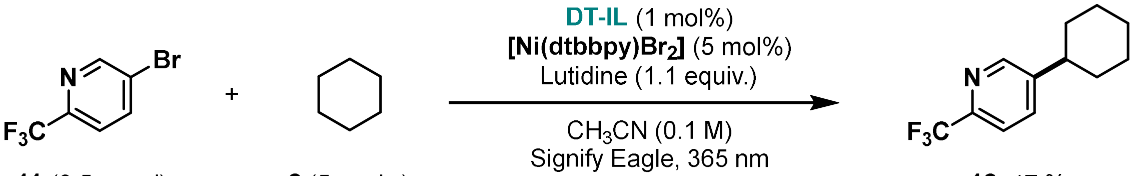

11 (0.5 mmol) **2** (5 equiv.) **12**, 47 %

To a flame-dried nitrogen-purged Schlenk flask, fitted with a rubber septum, IL-DT (22 mg, 0.005 mmol, 1 mol%), $NiBr_2$(dttbpy) (14 mg, 0.025 mmol, 5 mol%), 5-bromo-2-trifluoromethyl pyridine (**11**, 113 mg,

0.5 mmol, 1 equiv.) were added. The flask was evacuated and refilled with nitrogen three times. Dry and degassed acetonitrile (2.5 mL, 0.2 M) was added, and the mixture was sonicated to ensure complete dissolution. Cyclohexane (**2**, 301 µL, 2.5 mmol, 5 eq) was added before homogenizing. In another flask, Lutidine (64 µL, 0.55 mmol, 1.1 equiv.) was dissolved in dry and degassed acetonitrile (2.5 mL, 0.22 M).

These two solutions were taken separately with a 6 mL syringe (12.4 mm of diameter), positioned on a syringe pump and connected to a T-mixer. The latter was connected to the Signify reactor, set at 144 W. The two solutions were pumped through a 2.8 mL reactor with a residence time of 15 min and collected at the end of the reactor. The crude reaction mixture was extracted using the continuous extraction procedure, the crude product was obtained by evaporation of the combined pentane extractions, and purified by flash column chromatography (pentane 10:1 EtOAc).

^{1}H NMR (300 MHz, $CDCl_3$): 8.57 (s, 1H), 7.67 (dd, *J* = 8.1, 2.2 Hz, 1H), 7.60 (dd, *J* = 8.1, 0.8 Hz, 1H), 2.74 – 2.55 (m , 1H), 1.96 – 1.83 (m, 4H), 1.83 – 1.75 (m, 1H), 1.48 – 1.36 (m, 3H), 1.37 – 1.22 (m, 2H). ppm.

The NMR is in accordance with the literature.[7]

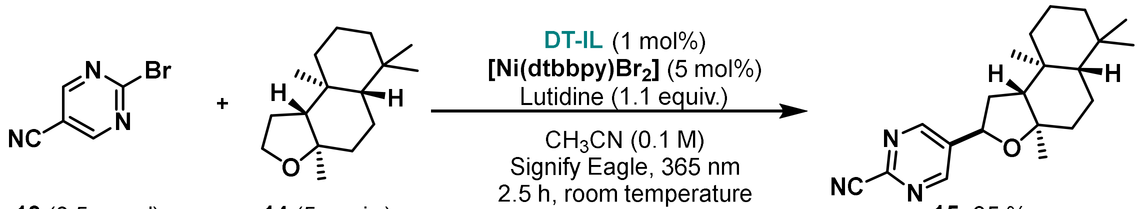

To a flame-dried nitrogen-purged Schlenk flask, fitted with a rubber septum, IL-DT (22 mg, 0.005 mmol, 1 mol%), $NiBr_2$(dttbpy) (14 mg, 0.025 mmol, 5 mol%), 5-bromo-2-cyano-pyridimidine (92 mg, 0.5mmol, 1 equiv.). The flask was evacuated and refilled with nitrogen three times. Dry and degassed acetonitrile (5 mL, 0.1 M) was added, and the mixture was sonicated to ensure complete dissolution. Ambroxide (591 mg, 2.5 mmol, 5 equiv.) was added before homogenizing. In another flask, Lutidine (64 µL, 0.55 mmol, 1.1 equiv.) was dissolved in dry and degassed acetonitrile (2.5 mL, 0.22 M).

These two solutions were taken separately with a 6 mL syringe (12.4 mm of diameter), positioned on a syringe pump and connected to a T-mixer. The latter was connected to the Signify reactor, set at 144 W. The two solutions were pumped through a 2.8 mL reactor with a residence time of 15 min and collected at the end of the reactor. The crude reaction mixture was extracted using the continuous extraction procedure, the crude product was obtained by evaporation of the combined pentane extractions, and purified by flash column chromatography (pentane 4:1 EtOAc) followed by recrystallization from heptane.

^{1}H NMR (300 MHz, CDCl$_3$): 8.81 (s, 2H), 5.22 (dd, *J* = 9.7, 2.5 Hz, 1H), 2.44 (ddd, *J* = 13.7, 11.8, 9.7 Hz, 1H), 2.08 (dt, *J* = 11.8, 3.2 Hz, 1H), 1.95 – 1.73 (m, 1H), 1.70 – 1.57 (m, 2H), 1.49 (dd, *J* = 13.7, 6.8 Hz, 1H), 1.45 – 1.34 (m, 2H), 1.33 – 1.23 (m, 9H), 0.91 – 0.86 (m, 9H), 0.84 (s, 3H) ppm.

Acid-based Minisci Coupling

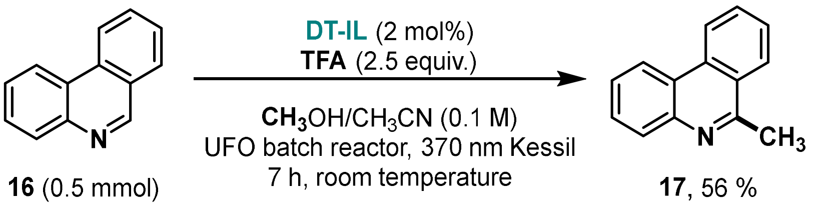

Acetonitrile (1 mL) and methanol (1 mL, 40 mmol, 200 equiv.) were added to P[6,6,6,14]DT (64 mg 0.015 mmol, 5 mol%) in a 7.5 mL glass vial, and the mixture was sonicated and shaken until complete dissolution of the catalyst (~1min). Phenanthridine (**16**, 53.8 mg, 0.3 mmol, 1 equiv.) was added to the vial along with a stir bar. TFA (38 µL, 0.5 mmol, 2.5 equiv.) was added to the reaction mixture, forming a precipitate. The vial was introduced in a "UFO" reactor for 7 h.

The mixture was quenched with a concentrated potassium carbonate solution, and the product is extracted twice with ethyl acetate. The organic extracts are combined and washed with brine, dried over anhydrous magnesium sulfate, and evaporated under reduced pressure. The crude reaction mixture was purified by flash column chromatography on silica gel to afford the pure methylated product.

Yield*: 22 mg (0.15 mmol, 56 %)

^{1}H-NMR (300 MHz, CDCl$_3$): δ = 8.61 (d, *J* = 6.8 Hz, 1H), 8.53 (dd, *J* = 8.1, 1.5 Hz, 1H), 8.21 (dd, *J* = 8.2, 1.4, 0.6 Hz, 1H), 8.14 (dd, *J* = 8.1, 1.4 Hz, 1H), 7.84 (ddd, *J* = 8.3, 7.1, 1.3 Hz, 1H), 7.77 – 7.67 (m, 2H), 7.67 – 7.59 (m, 1H), 3.06 (s, 3H) ppm.

5. Continuous Extraction Procedure

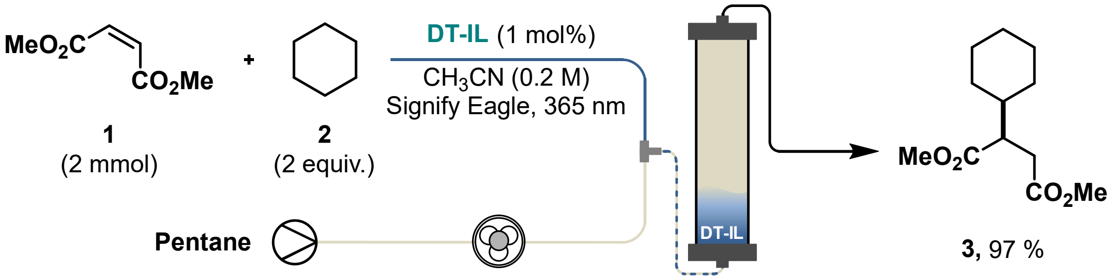

The crude reaction mixture was charged in an empty 10 g polypropylene chromatography cartridge. A peristaltic pump transferred pentane from a reservoir to the bottom of the cartridge with a flow rate of 5-8 mL/min, and the extraction flow was monitored by sampling and GC-MS, or if applicable by inline UV-Vis for the presence of the product. After the end of the extraction, the cartridge was opened, the pentane top layer was transferred to the combined pentane extract, and the oily deposit was redissolved in acetonitrile, filtered through a PTFE syringe filter, and evaporated under reduced pressure before characterization.*

* If needed, the recycled catalyst was placed in an oil bath at 130 °C under high vacuum to remove traces of organic impurities. The catalyst was shown by Marinetto and co-workers to be stable until 200 °C.[12]

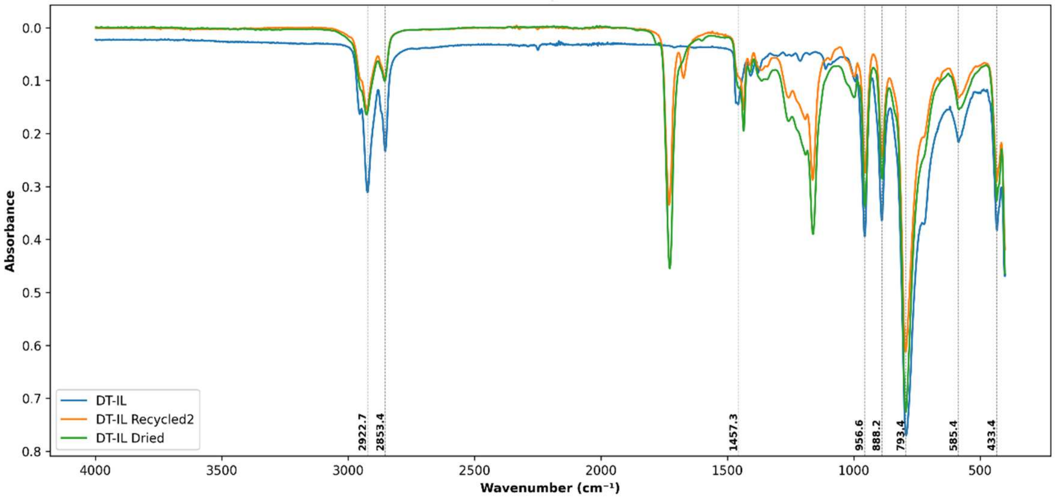

Figure S12. *FTIR of the DT-IL, reaction crude, and DT-IL after recycling and drying under high vacuum.*

After the recycling process, new peaks appear in the IR spectrum of the DT-IL catalyst, most likely corresponding to residual product. However, the catalytic activity remains unaffected, indicating that the DT-IL structure and functionality are preserved despite these minor spectral changes.

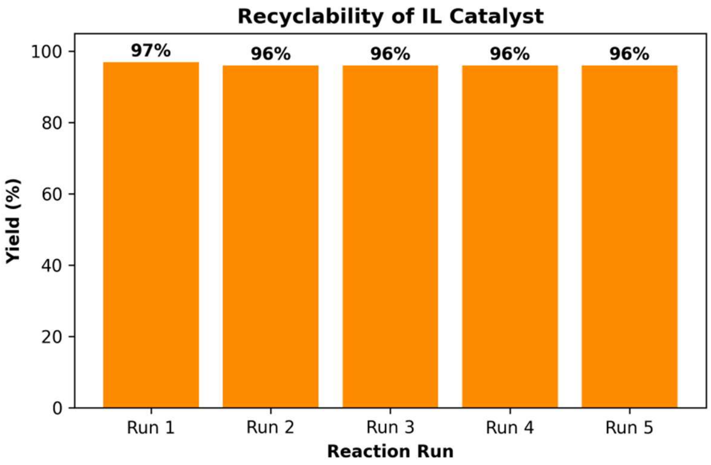

Figure S13. *Yield over run for the Giese-type coupling of cyclohexane with methyl malonate.*

6. Other Recycling Attempts

6.1. Recovery by precipitation

After performing a Giese reaction with tetrahydrofuran as an H donor, with a BDE of 92 kcal/mol for the C–H bond alpha to the oxygen, and solvent, we discovered that the addition of a liquid alkane (i.e., pentane/heptane) led to the separation of the mixture into a liquid phase and an oily deposit. This separation phenomenon was generally observed with any reaction solvent miscible with pentane (Figure S14). Preliminary experiments showed that adding fresh starting material to the oily phase and further irradiation afforded the product, indicating that the catalyst is still active after one cycle. However, the first issue we encountered was the increased solubility of the catalyst in the solvent/pentane mixture compared to pure pentane, which led to a low mass recovery of the catalyst in the oiled-out fraction.

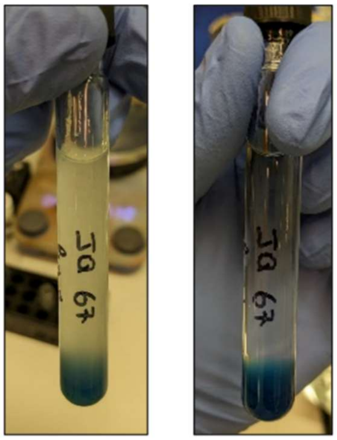

Figure S14*. Reaction mixture after the addition of pentane (left) and the same mixture after centrifugation (right).*

When performing the reaction under near solvent-free conditions, we realized the ionic liquid can also act as a good solvent and was found to trap part of the product within the oily residue. Furthermore, mixing the catalyst with the pentane layer for the extraction gets worse as the catalyst concentration increases. We attempted to mix the ionic liquid layer with acetone to reduce the viscosity between every pentane extraction, but this strategy led to an increase of the catalyst dissolved in the extracted pentane phase. It is possible to selectively recover a portion of the product by pentane extraction of the "on water" crude reaction mixture, but a large part of it is dissolved in the ionic liquid, forming a third phase (water-ionic liquid-pentane) with a poor mixing with the other phases as the catalyst is not soluble in either solvent.

6.2. Acid-base extraction

We then envisioned changing the strategy for an acid-base extraction. While it is generally accepted that the decatungstate POM is relatively sensitive to the pH of the medium, pH >2.5 leading to depolymerization,[8] we performed a proof of concept by extracting a mixture of cinnamic acid (**19**) (1.8 mmol) and ionic liquid catalyst (2 mol%) in DCM (0.3 M) with 1 M NaOH (pH=14). After evaporating the organic layer, the catalyst was recovered quantitatively and showed no sign of degradation. However, when performing the aerobic oxidation of alcohols (**18**) or aldehydes (Figure S15) with the ionic liquid catalyst in the absence of acid, we observed the formation of precipitates and loss of the catalyst. The reaction proceeded more efficiently in the presence of acid,[9] but adding a base during the extraction process led to the degradation of the catalyst.

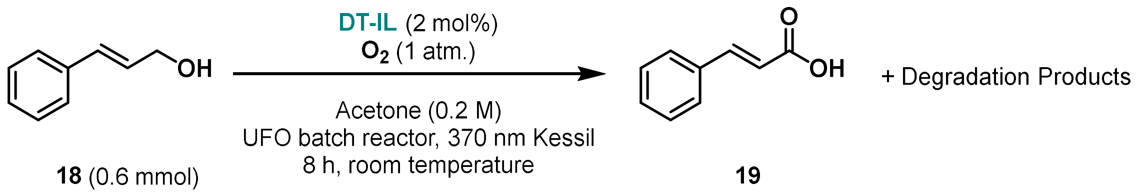

Figure S15. *Overall reaction scheme for the photocatalyzed aerobic oxidation of cinnamyl alcohol.*

The opposite strategy was envisioned with the formation of a basic substrate that could be extracted with acid. Multicomponent reactions to form amines by alkylation of in situ generated aryl imine (Figure S16) have been described for decatungstate catalysts.[10] Unfortunately, the crude reaction mixture contained unreacted starting material and byproducts in addition to the target amine, making the catalyst's recycling impossible by simple extraction. Performing a similar reaction with secondary alkyl amine[11] led to the formation of a blue precipitate that could arise from the formation of a cationic iminium intermediate (**20**) (Figure S16) that can interfere with the phosphonium ligands of our catalyst

or by acid/base reactivity on the reduced catalyst. We could not determine the exact nature of this precipitate, and this phenomenon was not mentioned in the literature.

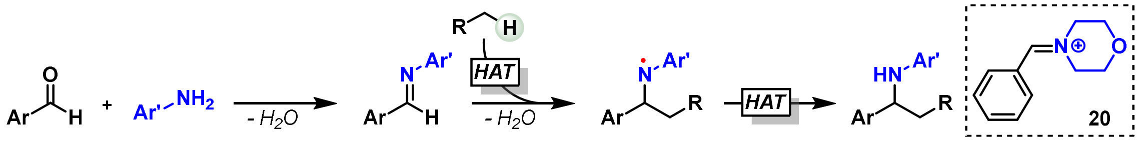

Figure S16. *Reaction scheme for the alkylation of in situ generated imines (left) and iminium intermediate (right).*

6.3. Chromatographic purification

Attempts at purifying the ionic liquid by polar chromatography (on silica or alumina) were unsuccessful. The ionic liquid catalyst typically has a significantly larger molecular weight (and thus size) than the starting material, product, and co-catalysts. It is possible to exploit this size difference using SEC. In this type of chromatography, the molecules smaller than the MWCO of the resin will interact with the resin pores, while the bigger molecule will elute with the solvent front without being retained. It is also advantageous that this method isn't sensitive to the polarity of the substrate, making it less substrate-dependent than other chromatography techniques. In the first report of the catalyst,[12] SEC was used to purify and recover the catalyst after use. Small-scale unsuccessful attempts were made to use SEC; however, this solution didn't appear very attractive for easy recycling as it becomes rapidly impractical, especially for large-scale reactions, as ideally, the column should be >100 times larger than the amount of material to process.

7. Kinetic Experiments

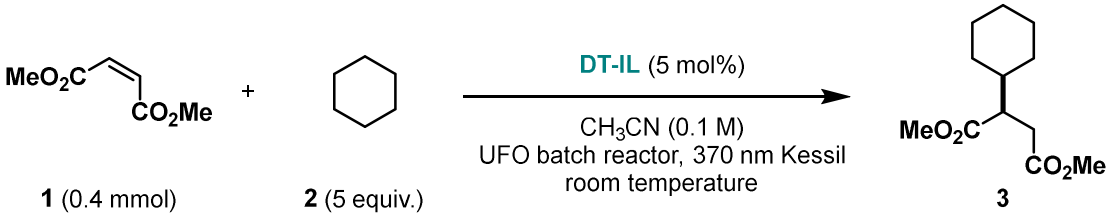

Acetonitrile (4 mL) was added to P[6,6,6,14]DT (85.7 mg, 0.02 mmol, 5 mol%) in a 7.5 mL glass vial, and the mixture was sonicated until complete dissolution of the catalyst (1 min). Dimethyl maleate (**1**, 50.1 mL, 0.4 mmol, 1 equiv.) and cyclohexane (**2**, 216 mL, 2.0 mmol, 5 equiv.) were added to the vial along with a stir bar. The mixture was shaken and introduced in the "UFO" reactor. 100 mL aliquots were taken after 0.5, 1, 2, 3 and 4 h (reactor switch off during sampling) and 1 equiv. of CH_2Br_2 was added as internal standard. Yields were calculated by comparing the internal standard (s, 2H, 4.90 ppm) with the methyl signals of SM (s, 3H, 3.70 ppm) and product (s, 3H, 3.59 and s, 3H, 3.57 ppm).

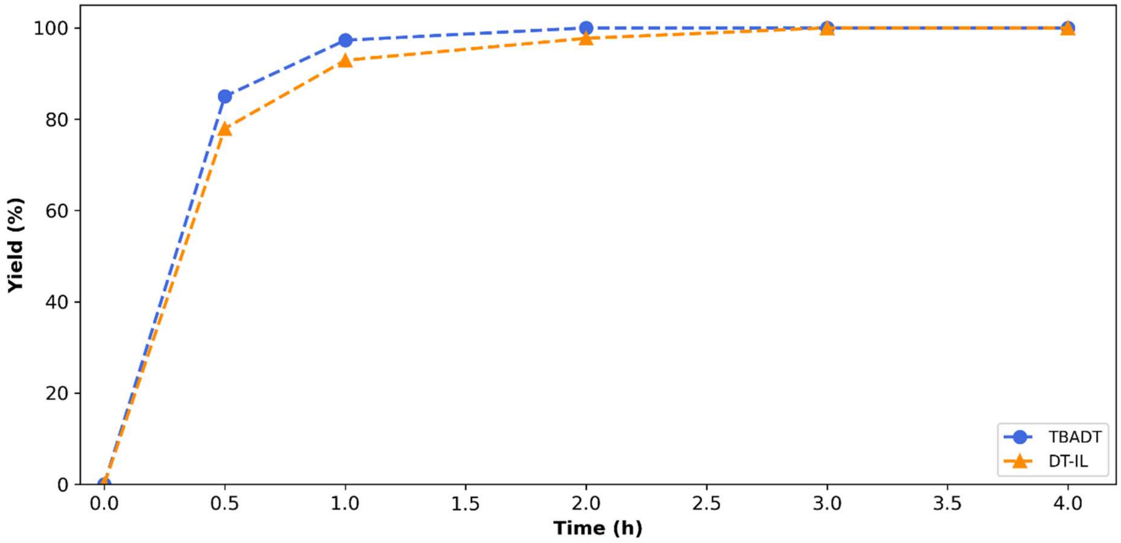

Figure S17. *Comparison of the similar behavior of TBADT and DT-IL for the Giese type coupling of cyclohexane and dimethyl maleate.*

8. Solvent Screening

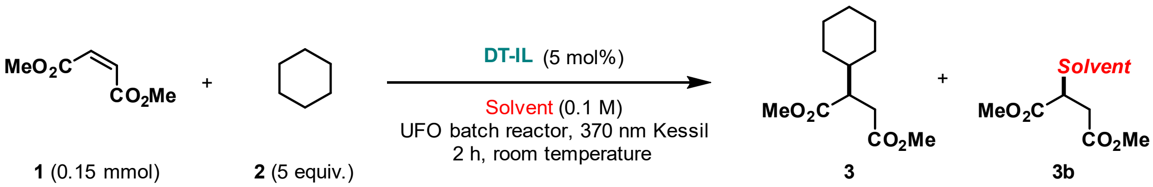

Desired solvent (1.5 mL) was added to P[6,6,6,14]DT (32.2 mg, 0.0075 mmol, 5 mol%) in a 7.5 mL glass vial, and the mixture was sonicated until complete dissolution of the catalyst (1 min). Dimethyl maleate (**1**, 18.8 mL, 0.15 mmol, 1 equiv.) and cyclohexane (**2**, 81.1 mL, 0.75 mmol, 5 equiv.) were added to the vial along with a stir bar. The mixture was shaken and transferred into the "UFO" reactor, where it was stirred for 2 hours under irradiation with a 370 nm Kessil lamp. After said time, 1 equiv. of CH_2Br_2 was added as internal standard. Yields were calculated by comparing the internal standard (s, 2H, 4.90 ppm) with the methyl signals of SM (s, 3H, 3.70 ppm) and product (s, 3H, 3.59 and s, 3H, 3.57 ppm).

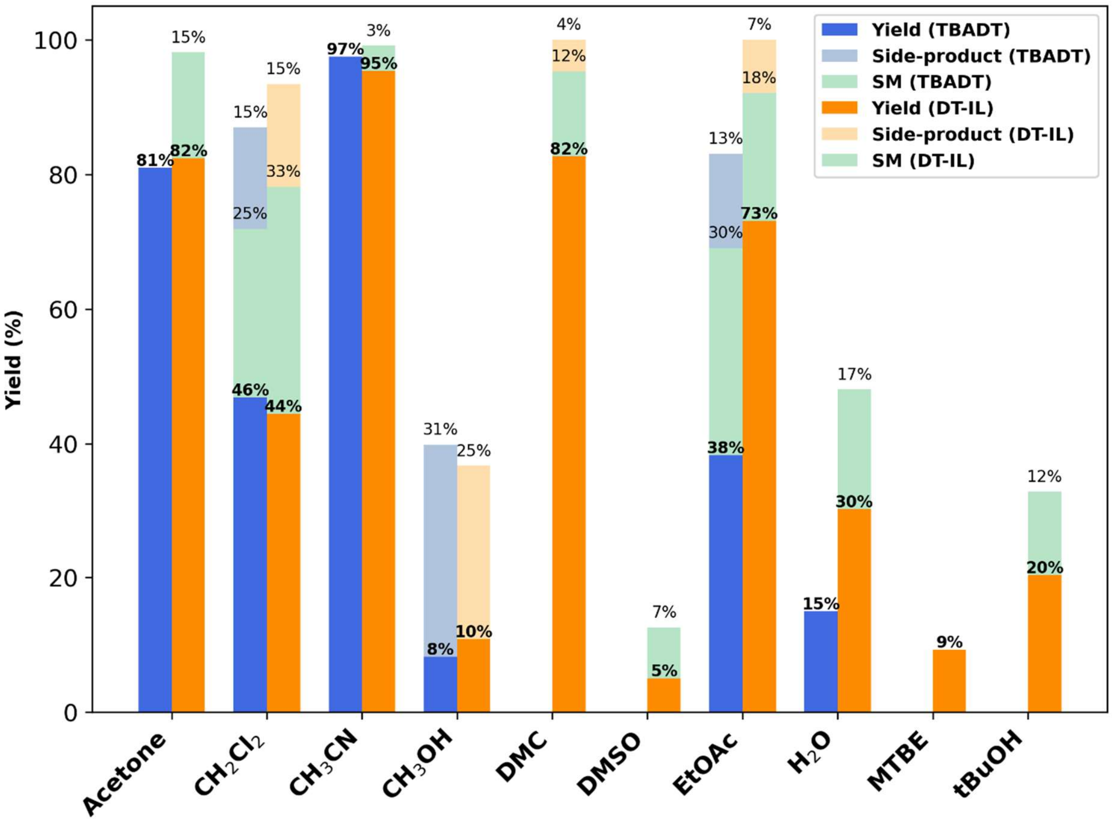

Figure S18. *Comparison of Yield, SM and side product percentages of the Giese-type coupling of cyclohexane and methyl maleate with different catalyst and solvents.*

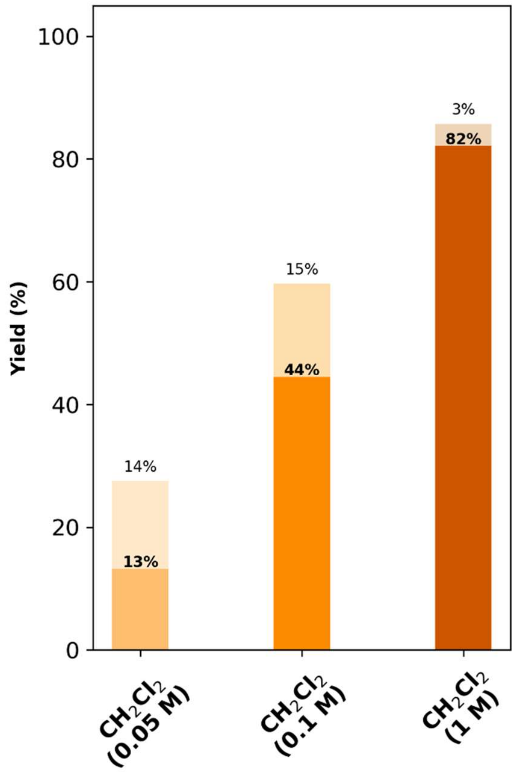

Figure S19. *Comparison of Yield, SM and side product percentages of the Giese-type coupling of dimethyl maleate (**1**) and cyclohexane (**2**) with different concentrations of the dimethyl maleate in DCM. Ratio DCM/Cyclohexane; 1 M (15.7 mmol/ 5 mmol); 0.1 M (78.5 mmol/0.5 mmol); 0.05 M (78.5 mmol/0.25 mmol).*

9. E Factor Calculations

The E-factor (Environmental Factor) is a metric in green chemistry introduced by Roger Sheldon[13] that measures the amount of waste generated per amount of product obtained (kg of waste per kg of product). A lower E-factor indicates a more environmentally friendly and sustainable process, as it reflects higher efficiency and reduced environmental impact.

Industry / Process Type	Typical E-Factor
Oil refining	< 0.1
Bulk chemicals	1–5
Fine chemicals	5–50
Pharmaceuticals	25–100+

$$E_{factor} = \frac{total\ mass\ of\ inputs\ - mass\ of\ product}{m(Product)}$$

"Neat" Giese Coupling

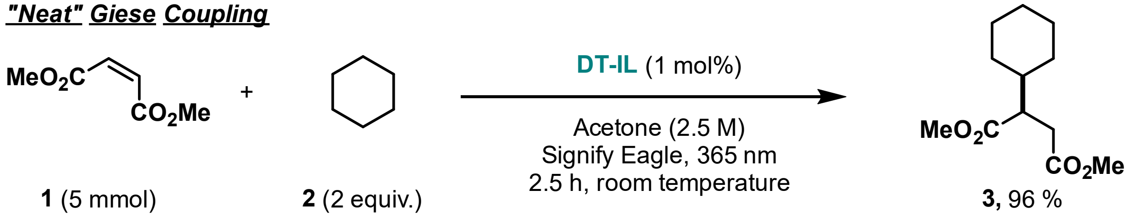

$$E(neat) = \frac{[m(SM) + m(C-H) + m(Catalyst) + V(Solvent) * d(Solvent)] - m(Product)}{m(Product)}$$

$$= \frac{\left[721\,mg + 850\,mg + 214\,mg + 300\,uL * 0.784\frac{mg}{uL}\right] - 1095\,mg}{1095\,mg} = \mathbf{0.85}$$

By taking out the catalyst mass, as has being fully recovered;

$$E(neat + catalyst\ recycling) = \frac{[m(SM) + m(C-H) + V(Solvent) * d(Solvent)] - m(Product)}{m(Product)} =$$

$$= \frac{\left[721\,mg + 850\,mg + 300\,uL * 0.784\frac{mg}{uL}\right] - 1095\,mg}{1095\,mg} = \mathbf{0.65}$$

For comparison, E-factors were also calculated for standard Giese coupling conditions using either IL or TBADT as the catalyst with 5 equivalents of cyclohexane and 0,1 M concentration of the starting material.

Giese Coupling

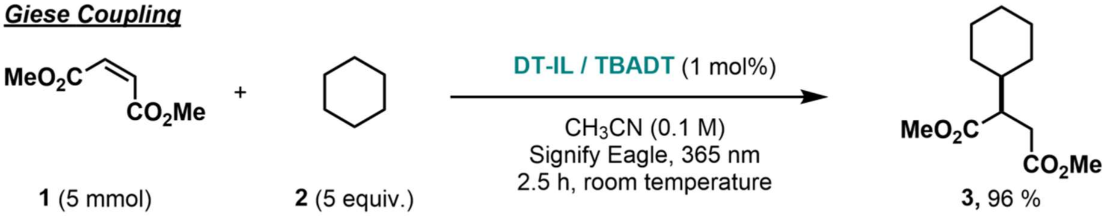

$$E(\text{Giese}) = \frac{[m(Reagents) + m(Solvent)] - m(Product)}{m(Product)}$$

$$E(\text{Giese\_IL}) = \frac{[m(SM) + m(C-H) + m(Catalyst) + V(Solvent) * d(Solvent)] - m(Product)}{m(Product)}$$

$$= \frac{\left[43\,mg + 125\,mg + 3000\,uL * 0.789\frac{mg}{uL}\right] - 65\,mg}{65\,mg} = \mathbf{38.2}$$

$$E(\text{Giese\_TBADT}) = \frac{[m(SM) + m(C-H) + m(Catalyst) + V(Solvent) * d(Solvent)] - m(Product)}{m(Product)}$$

$$= \frac{\left[43\,mg + 125\,mg + 3000\,uL * 0.789\frac{mg}{uL}\right] - 66\,mg}{66\,mg} = \mathbf{37.6}$$

"On-Water" reaction

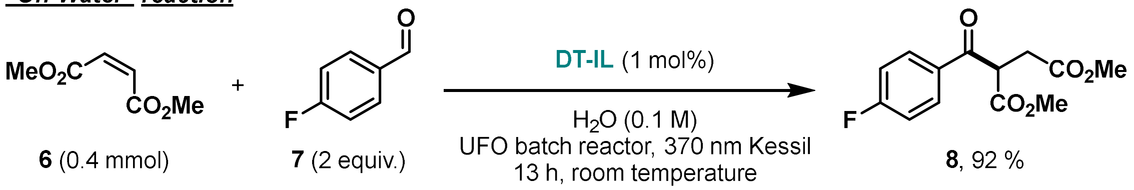

In the case of the "On-Water" reaction, the E-factor was calculated as the total mass of inputs per mass of product excluding water. In line with the guidelines proposed by Sheldon,[14] if the total amount of solvent leaving the process is not known, it is assumed that the solvent is recycled with a 10% loss considered as waste. This assumption is particularly applicable here, as the reaction proceeds without organic solvents, achieves a 92% yield, and requires only 2 equivalents of the coupling partner. Furthermore, the catalyst is immiscible with water, allowing for straightforward separation and enabling the recyclability of both the catalyst and water. Under these conditions, a 90% recovery of water is considered realistic and aligns with practices in which water can be reused in subsequent reactions after minimal treatment. For completeness, we also report the E-factor considering all water as waste to provide both conservative and more process-representative values.

<u>E-Factor of the "On-Water" reaction considering all the water as waste</u>

$$E(\text{On water} + catalyst\ recycling) = \frac{[m(Reagents) + m(Solvent)] - m(Product)}{m(Product)}$$

$$= \frac{[m(SM) + m(C-H) + V(Solvent) * d(Solvent)] - m(Product)}{m(Product)} =$$

$$= \frac{\left[57\,mg + 100\,mg + 4000\,uL * 1.00\frac{mg}{uL}\right] - 94\,mg}{94\,mg} = \mathbf{43.2}$$

<u>E-Factor of the "On-Water" reaction considering the recovery of 90% of the water</u>

$$E(\text{On water} + catalyst\ recycling + solvent\ recycling)$$

$$= \frac{[m(Reagents) + 0.1\ x\ m(Solvent)] - m(Product)}{m(Product)}$$

$$= \frac{[m(SM) + m(C-H) + V(Solvent) * d(Solvent)] - m(Product)}{m(Product)} =$$

$$= \frac{\left[57\,mg + 100\,mg + 0.1\ x\ 4000\,uL * 1.00\frac{mg}{uL}\right] - 94\,mg}{94\,mg} = \mathbf{4.92}$$

10. References

1. T. M. Masson, S. D. A. Zondag, J. H. A. Schuurmans, and T. Noël, *React. Chem. Eng.*, 2024, **9**, 2218–2225.
2. T. Wan, Z. Wen, G. Laudadio, L. Capaldo, R. Lammers, J. A. Rincón, P. García-Losada, C. Mateos, M. O. Frederick, R. Broersma and T. Noël, *ACS Cent. Sci.*, 2022, **8**, 51–56.
3. Y. Wang, M. Wang, H. Wang, X. Li, and H. Jiang, *Adv. Synth. Catal.*, 2015, **357**, 2644–2650.
4. G. Laudadio, Y. Deng, K. van der Wal, D. Ravelli, M. Nuño, M. Fagnoni, D. Guthrie, Y. Sun, and T. Noël, *Science*, 2020, **369**, 92–96.
5. M. A. Maskeri, M. L. Schrader and K. A. Scheidt, *Angew. Chem. Int. Ed.*, 2022, **61**, e202117002.
6. Y. Wang, M. Wang, H. Wang, X. Li, and H. Jiang, *Adv. Synth. Catal.*, 2015, **357**, 2644–2650.
7. D. Mazzarella, A. Pulcinella, L. Bovy, R. Broersma, and T. Noël, *Angew. Chem. Int. Ed.*, 2021, **60**, 21277–21282.
8. E. Papaconstantinou, A. Hiskia, and A. Troupis, *Faraday Discuss.*, 2003, **8**, 813–825.
9. G. Laudadio, S. Govaerts, Y. Wang, D. Ravelli, H. F. Koolman, M. Fagnoni, S. W. Djuric, and T. Noël, *Angew. Chem. Int. Ed.*, 2018, **57**, 4078–4082.
10. S. Pillitteri, P. Ranjan, L. G. Voskressensky, E. V. Van der Eycken, and U. K. Sharma, *Mol. Catal.*, 2021, **514**, 111841.
11. R. Kumar, N. J. Flodén, W. G. Whitehurst, and M. J. Gaunt, *Nature*, 2020, **581**, 415–420.
12. Y. Martinetto, B. Pégot, C. Roch-Marchal, M. Haouas, B. Cottyn-Boitte, F. Camerel, J. Jeftic, D. Morineau, E. Magnier, and S. Floquet, *New J. Chem.*, 2021, **45**, 9751–9755.
13. a) R. A. Sheldon, *Chem. Ind., 1992*, 903–906 b) R. A. Sheldon, *Green Chem., 2017*, **19**, 18-43.
14. R. A. Sheldon, *Green Chem., 2023*, **25**, 1704-1728.

11. Spectra

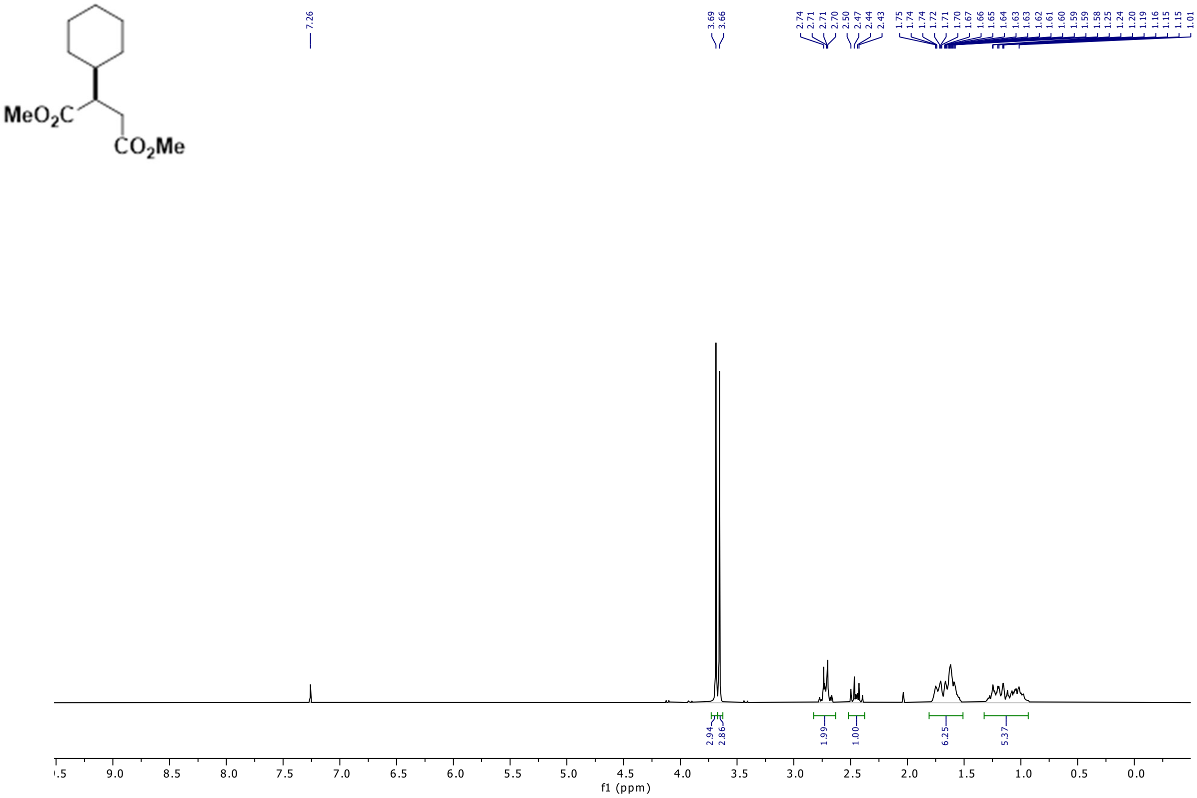

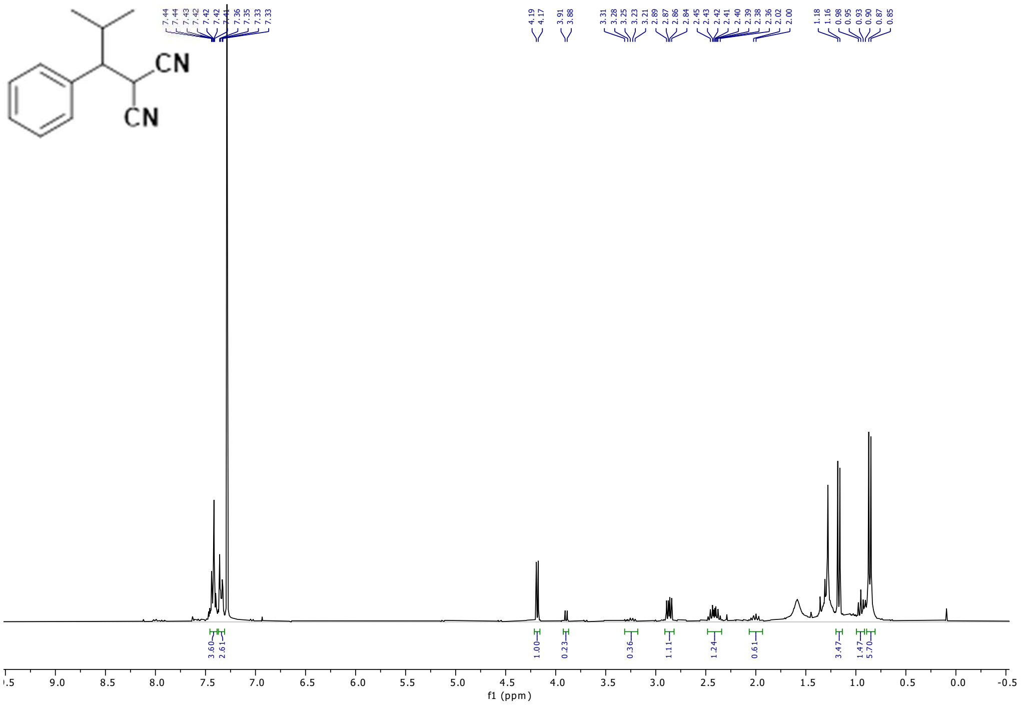

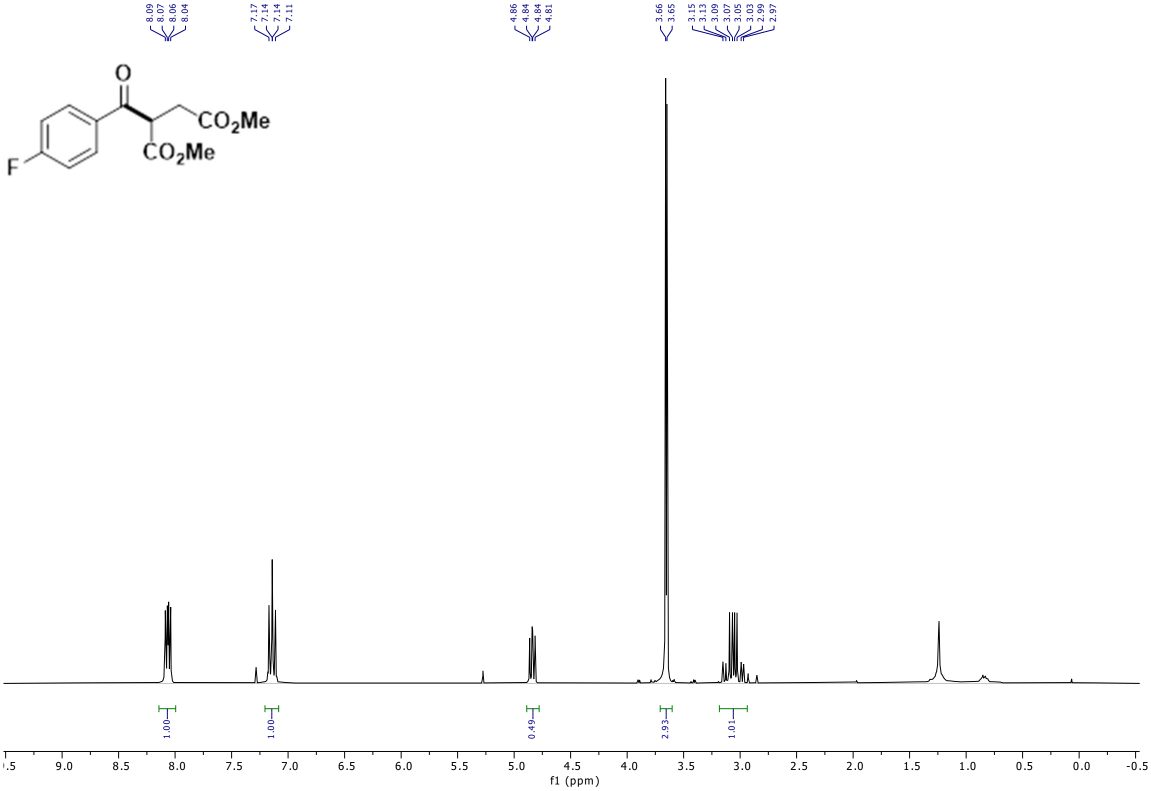

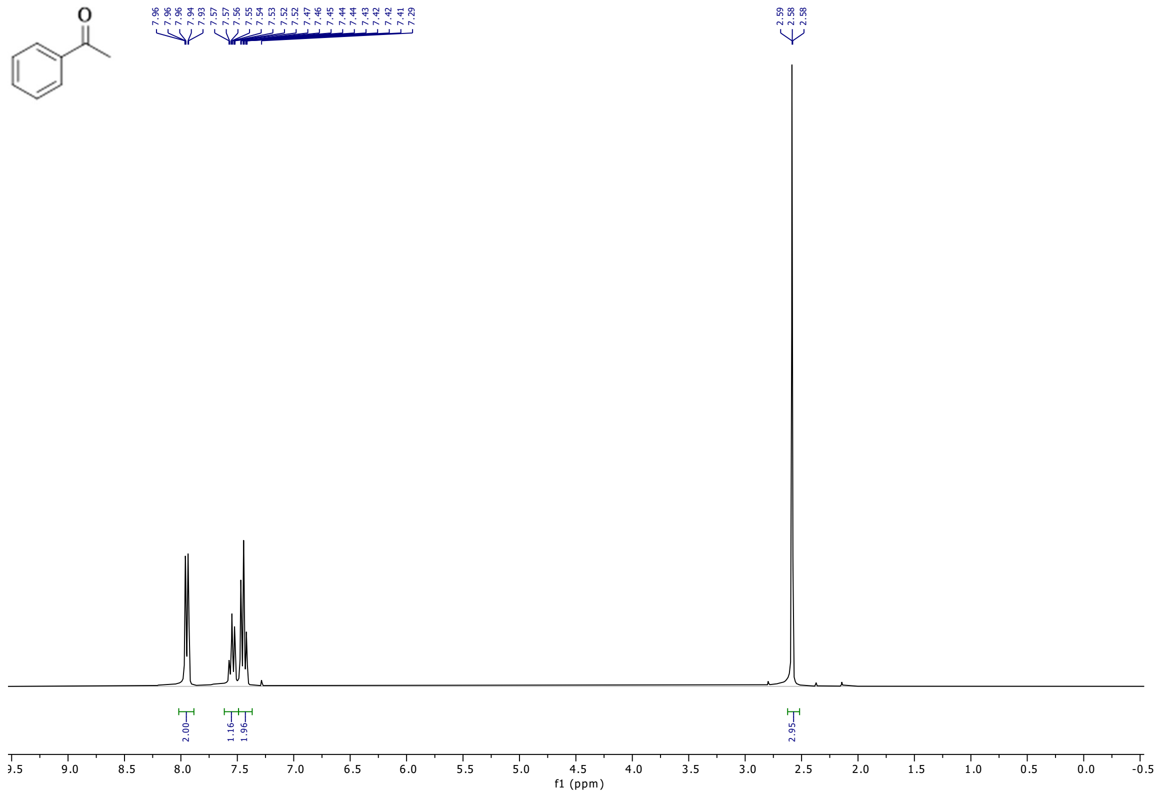
O
7.96
7.96
7.96
7.94
7.93
7.57
7.57
7.56
7.55
7.54
7.53
7.52
7.52
7.47
7.46
7.45
7.44
7.44
7.43
7.42
7.42
7.41
7.29
2.59
2.58
2.58
2.00
1.16
1.96
2.95
9.5 9.0 8.5 8.0 7.5 7.0 6.5 6.0 5.5 5.0 4.5 4.0 3.5 3.0 2.5 2.0 1.5 1.0 0.5 0.0 -0.5
f1 (ppm)

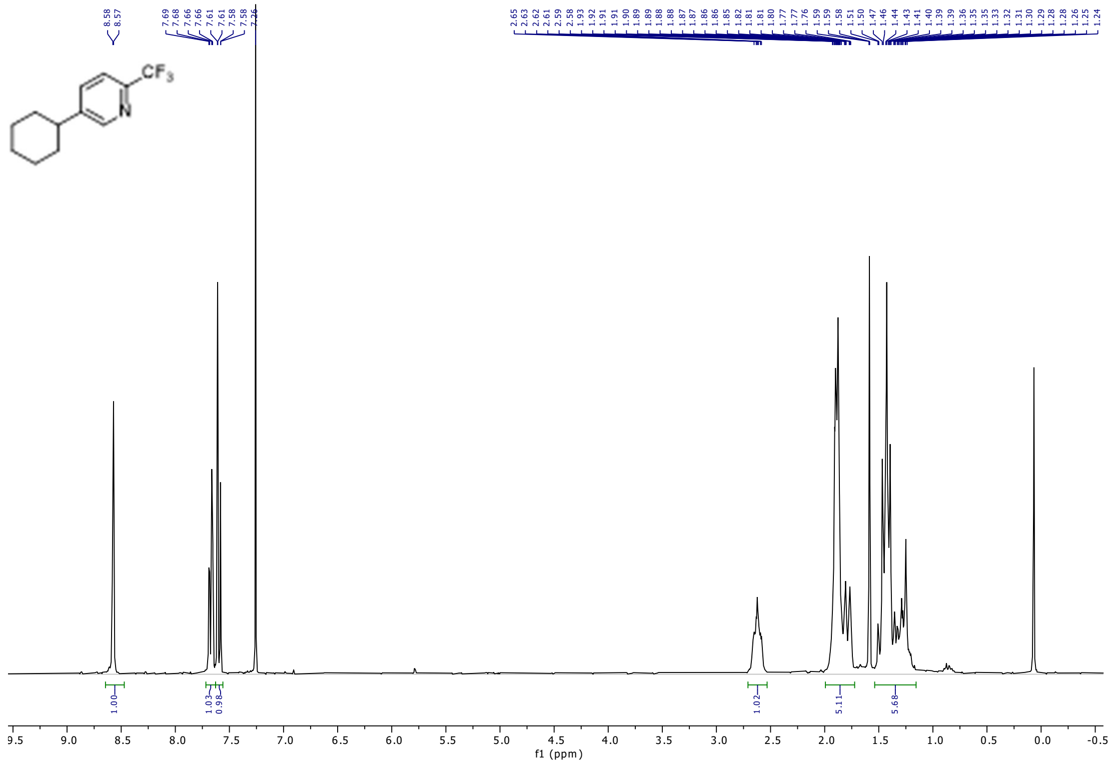
CF3
N
8.58
8.57
7.69
7.68
7.66
7.66
7.61
7.58
7.58
7.26
2.65
2.63
2.62
2.61
2.59
2.58
1.93
1.92
1.91
1.91
1.90
1.89
1.88
1.88
1.87
1.87
1.86
1.85
1.82
1.81
1.81
1.80
1.77
1.76
1.59
1.59
1.58
1.51
1.50
1.47
1.46
1.44
1.43
1.41
1.40
1.39
1.39
1.36
1.35
1.35
1.33
1.32
1.31
1.30
1.29
1.28
1.28
1.26
1.25
1.24
1.00
1.03
0.98
1.02
5.11
5.68
9.5 9.0 8.5 8.0 7.5 7.0 6.5 6.0 5.5 5.0 4.5 4.0 3.5 3.0 2.5 2.0 1.5 1.0 0.5 0.0 -0.5
f1 (ppm)

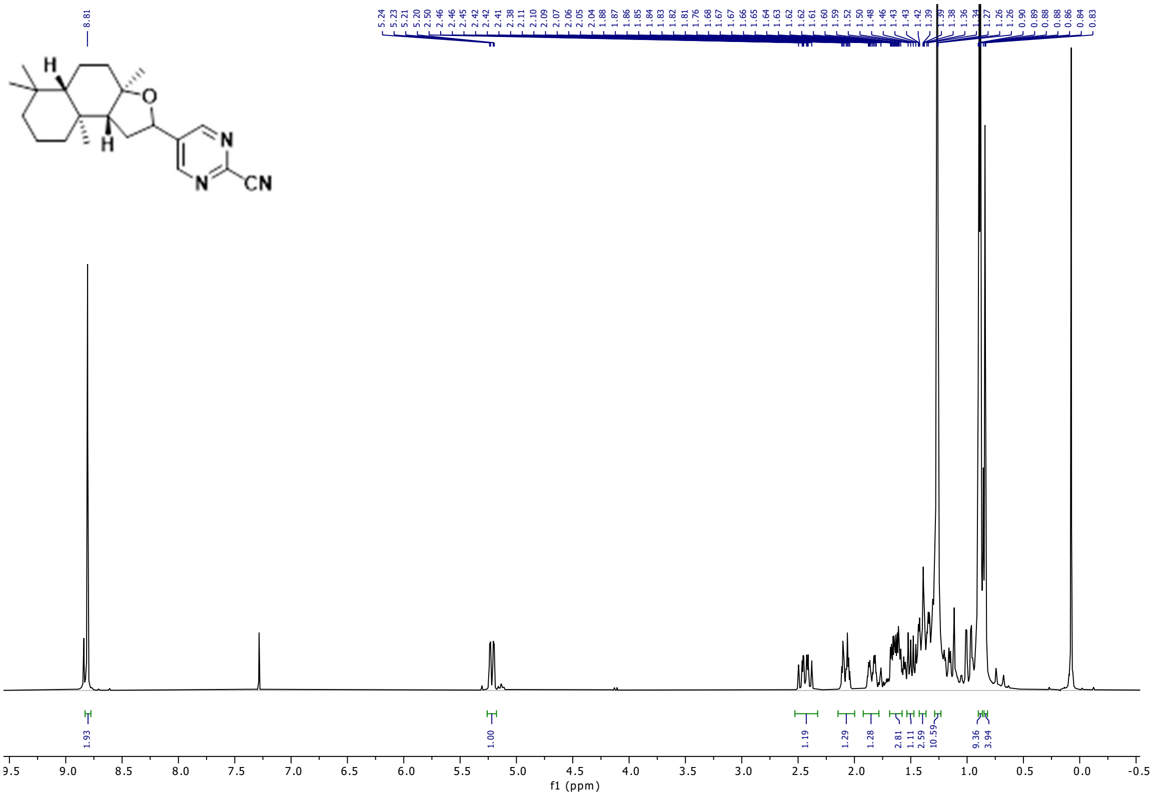
f1 (ppm)

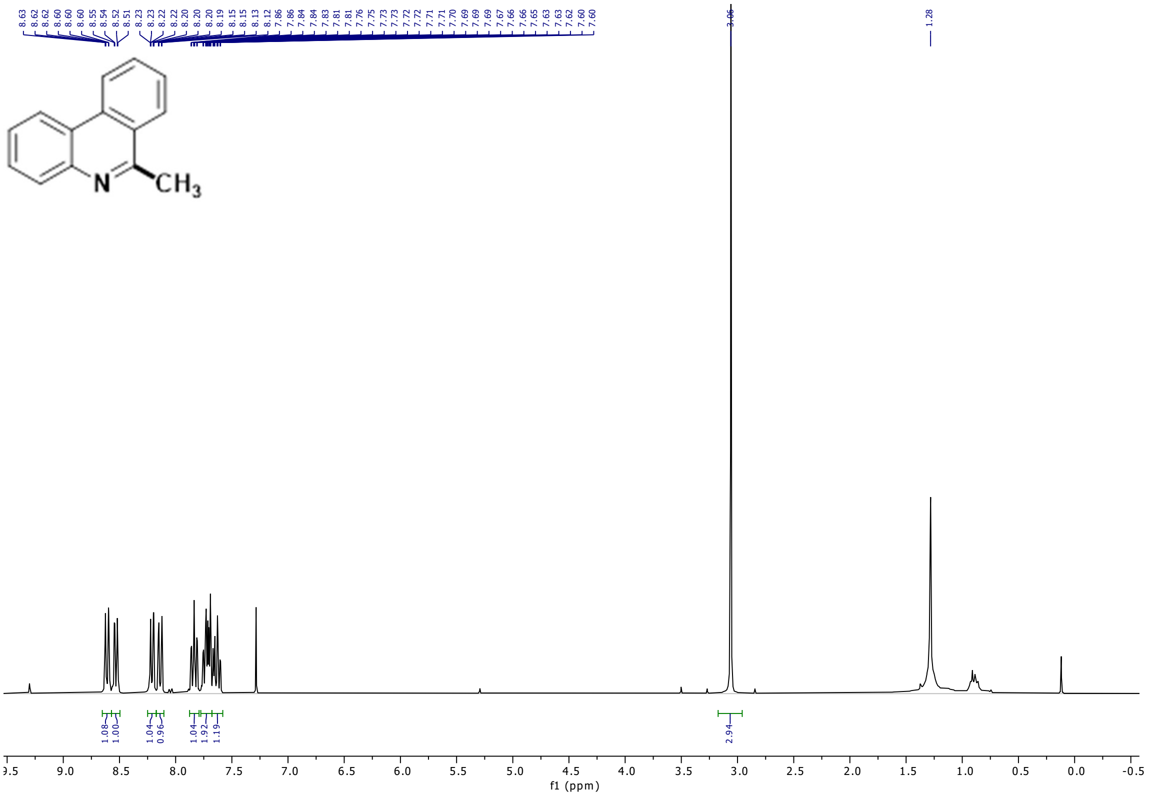
f1 (ppm)
